# The Difficult Marriage of Triarylcorroles with Zinc and Nickel Ions

**DOI:** 10.1021/acs.inorgchem.2c03099

**Published:** 2022-10-26

**Authors:** Mario L. Naitana, W. Ryan Osterloh, Lorena Di Zazzo, Sara Nardis, Fabrizio Caroleo, Pierluigi Stipa, Khai-Nghi Truong, Kari Rissanen, Yuanyuan Fang, Karl M. Kadish, Roberto Paolesse

**Affiliations:** † Department of Chemical Science and Technologies, University of Rome Tor Vergata, Via della Ricerca Scientifica, 00133Roma, Italy; ‡ Department of Chemistry, University of Houston, Houston, Texas77204-5003, United States; § Dipartimento di Scienze e Ingegneria della Materia, dell’Ambiente ed Urbanistica, Università Politecnica delle Marche, Via Brecce Bianche 12, 60131Ancona, Italy; ∥ Department of Chemistry, University of Jyväskylä, 40014Jyväskylä, Finland

## Abstract

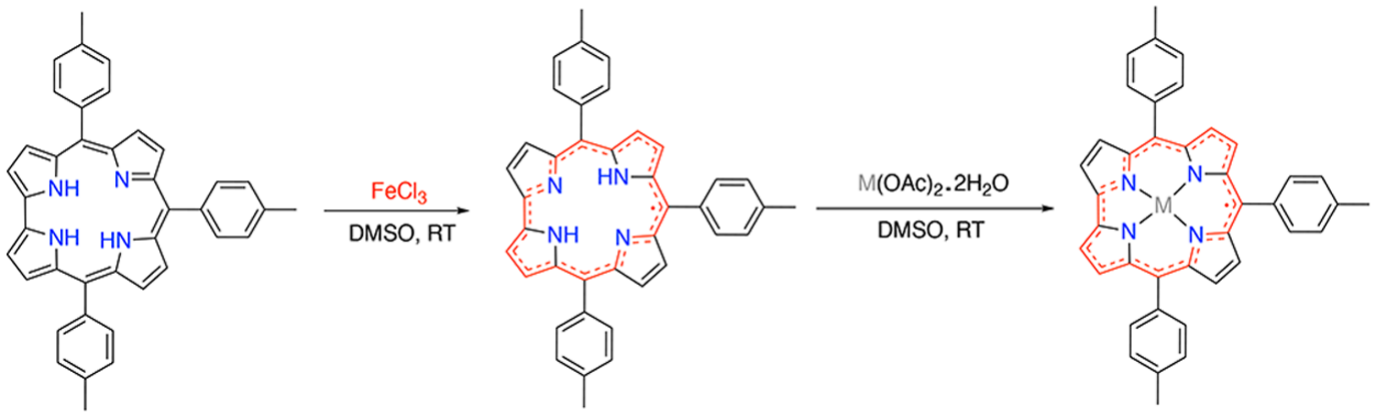

The coordination chemistry of corrole has witnessed a great improvement in the past few years and its Periodic Table has been widened to be so large that it is compared with that of porphyrins. However, Ni and Zn ions, commonly used with porphyrins for both synthetic and theoretical purposes, are sparsely reported in the case of corroles. Here, we report synthetic protocols for preparing Ni and Zn triarylcorrole complexes. In the case of Zn, the preliminary oxidation of the free base corrole in DMSO to the neutral corrole radical is a necessary step to obtain the coordination of the metal ion, because the direct reaction led to the formation of an open-chain tetrapyrrole. The Ni complex could be directly obtained by heating the free base corrole and Ni(II) salt to 100 °C in a DMSO solution containing FeCl_3_. The non-innocent nature of the corrole ligand for both complexes has been elucidated by EPR, and in the case of the Zn derivative the first spectroelectrochemical characterization is presented.

## Abstract 2

The oxidation of triarylcorrole to the corresponding neutral radical species in DMSO is a key step to allow the preparation of the corresponding Ni and Zn complexes. Without this step, the oxidative ring opening of the macrocycle occurs, leading to the formation of a linear tetrapyrrole. The spectroscopic characterization of these species indicates their radical character. The stability of the Zn complex can be improved by peripheral substitution.

## Introduction

The 60th anniversary marking the first reported corrole preparation is approaching,^1^ but this macrocycle has only assumed a leading role in the porphyrinoid family in the last two decades. In the past, the lengthy preparation of β-alkylcorroles^2^ prevented widespread investigations of corrole macrocycles, while the more recent discovery of viable synthetic pathways for larger scale preparation of triarylcorroles from commercial precursors^3^ permitted more abundant and detailed studies of corrole complexes and their physicochemical properties.

The rising interest in corroles derives from their multifaceted properties, which make the chemistry of this macrocycle different from the parent porphyrins and consequently interesting for both theoretical and practical studies.^4^ The contrasting coordination chemistry of metallocorroles compared to related porphyrins is a perfect example. This difference in behavior between the 23 and 24 atom tetrapyrrole macrocycles stems from the particularly challenging interpretation of metallocorrole electronic structures as opposed to the straightforward assignment of metal and ligand oxidation state for porphyrin complexes,^5^ a property which makes corrole derivatives of interest for catalytic applications.^6^

Both the trianionic character and contracted nature of the corrole macrocycle make these compounds electron rich with strong σ-donor character, thus leading to facile corrole-centered oxidation potentials. However, these characteristics allow for a markedly facile ligand-to-metal electron transfer, resulting in the non-innocent character of the corrole ligand and, therefore, often complicating an elucidation of the metallocorrole electronic structure (*i.e.*, metal oxidation state). This difficulty has, in the past, sometimes resulted in a series of noncorrect characterizations of corrole complexes.^7^

The iron, cobalt, and copper corrole complexes are examples of compounds whose properties have been reexamined and reviewed multiple times over the years, owing to the ambiguity between their “innocent” and “non-innocent” behavior, a phenomenon which was found to be modulated not only by external physical conditions (e.g., temperature) but also by the chemical nature of the substituents on the periphery of the macrocycle as well as by the type and number of axial ligand(s) coordinated to the central metal ion.^8−10^

Despite the deceptive nature of their electronic structure, a great deal of research has led to an expanded Periodic Table of metallocorrolates, which is beginning to rival that of the corresponding porphyrins. Surprisingly, derivatives with nickel and zinc central metal ions, widely utilized in the porphyrin field, have been essentially ignored in the case of corroles owing, in part, to the mismatch among the preferred divalent charge of these metal ions and the trianionic character of the corrole; however, it should be noted that this charge mismatch has not influenced the preparation of numerous copper corrolates, which have been tested to explore the possible demetalation of metallocorrolates.^11^

While nickel corroles were among the first reported examples of metal octaalkylcorroles,^12^ the characterization of these complexes also provided one of the first examples for the occasionally deceptive nature of the metallocorrole electronic structure. In fact, nickel corroles were initially characterized as a dianionic nonaromatic isocorrole species with a hydrogenated sp^3^*meso*-carbon at the 10 position or as a partially deprotonated corrole complex,^13^ the latter of which was consistent with the reactivity of the complex with bases. Years later, the same nickel octaalkylcorrole was characterized by Vogel and co-workers^14^ as a non-innocent system having a neutral Ni(II) central metal ion and an oxidized corrole radical ligand. However, a detailed characterization of the nickel triarylcorrole is still absent, with only a few short reports in the literature.^10,15^ Very recently, Gross and co-workers have reported the preparation of some anionic Ni complexes based on the electron poor 5,10,15-tris(pentafluophenyl)corrole (H_3_TPFCorr) as suitable catalysts for the hydrogen evolution reaction.^16^ In this case, the anionic complex was obtained from the metalation reaction carried out in DMF or pyridine and the β-substitution with electron-withdrawing substituents was necessary to stabilize the anionic complexes, because the bare [Ni(TPFCorr)]^−^ was unstable in noncoordinating solvents. The definition of an optimized route for the preparation and characterization of the neutral Ni corrole is still achievable.

In the case of zinc corroles, the literature is even more sparse. The first report of a zinc corrole described an anionic Zn(II) octamethylcorrole complex obtained in pyridine,^17^ while formally dianionic *N*-alkylated corroles have been shown to easily coordinate zinc ions as observed in the case of dianionic porphyrins. In 2015, Bröring and co-workers were able to isolate the air-stable 3,17-dichloro-5,10,15-trimesitylcorrole radical, H_2_(3,17-Cl_2_Corr^•^), from the reaction of 5,10,15-trimesitylcorrole [H_3_(Corr)] with tungsten hexachloride and tungsten hexacarbonyl.^18^ Attempts to metalate this free base corrole radical with zinc(II) acetate dihydrate [Zn(OAc)_2_·2H_2_O] led to formation of the first zinc triarylcorrole complex showing the foregone non-innocent behavior of the macrocycle (*i.e.*, a complex possessing a Zn(II) central metal ion and a corrole π-cation radical, Zn^II^(Cor^•^)). Moreover, the molecular design of this complex benefitted from the bulky mesityl groups that helped minimize aggregation and oligomerization in solution, while the two electron-acceptor β-chlorine substituents increased the radical character of the corrole ligand, thus leading to stabilization of the Zn(II) complex.

Although both nickel and zinc corrole complexes have now been characterized as non-innocent derivatives possessing a corrole π-cation radical, a complete description of the coordination behavior of these species has not yet been reported. This is now described on the following pages where we report the synthesis, spectroscopic, and electrochemical characterization of the products obtained from the reaction of trianionic triarylcorroles with divalent nickel and zinc salts.

## Results and Discussion

### Synthesis and Characterization of Zinc Corroles

In the current study, metalation with zinc and nickel were first attempted using two meso-aryl substituted corroles, namely, 5,10,15-triphenylcorrole [H_3_(TPCorr)] and 5,10,15-tritolylcorrole [H_3_(TTCorr)]. These core corrole structures lacking sterically bulky functional groups were purposefully chosen to allow possible dimerization/oligomerization indicative of the generated zinc and/or nickel corrole complexes possessing a macrocycle centered radical.

**Scheme 1:**
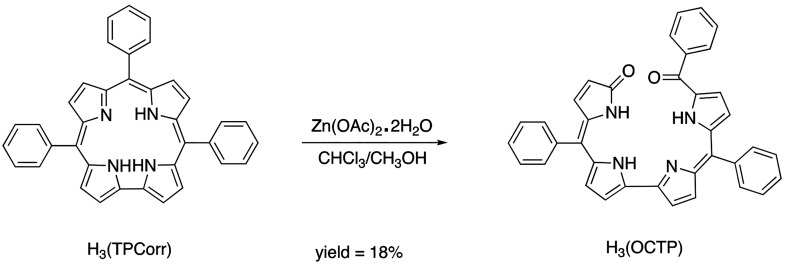
Synthetic Pathway for the Formation of an Open-Chain Linear Tetrapyrrole from a Reaction of H_3_(TPCorr) with Zn(OAc)_2_·2H_2_O

**Figure 1:**
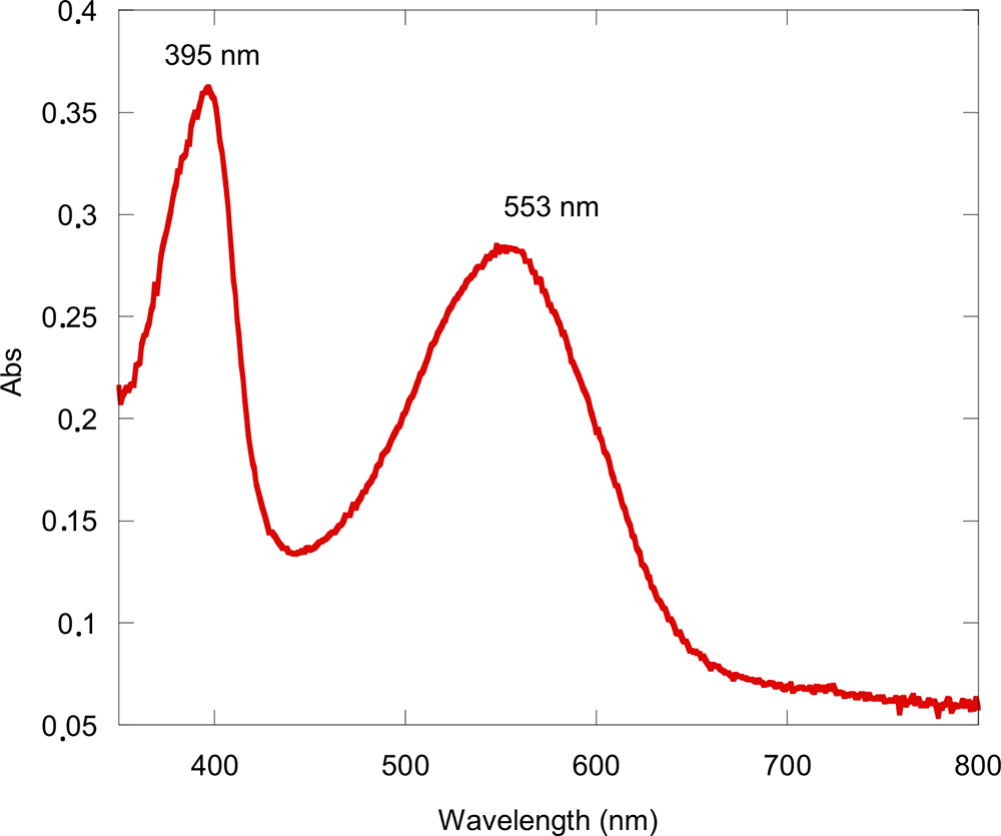
UV–visible spectrum of H_3_(OCTP) in CH_2_Cl_2_.

**Figure 2:**
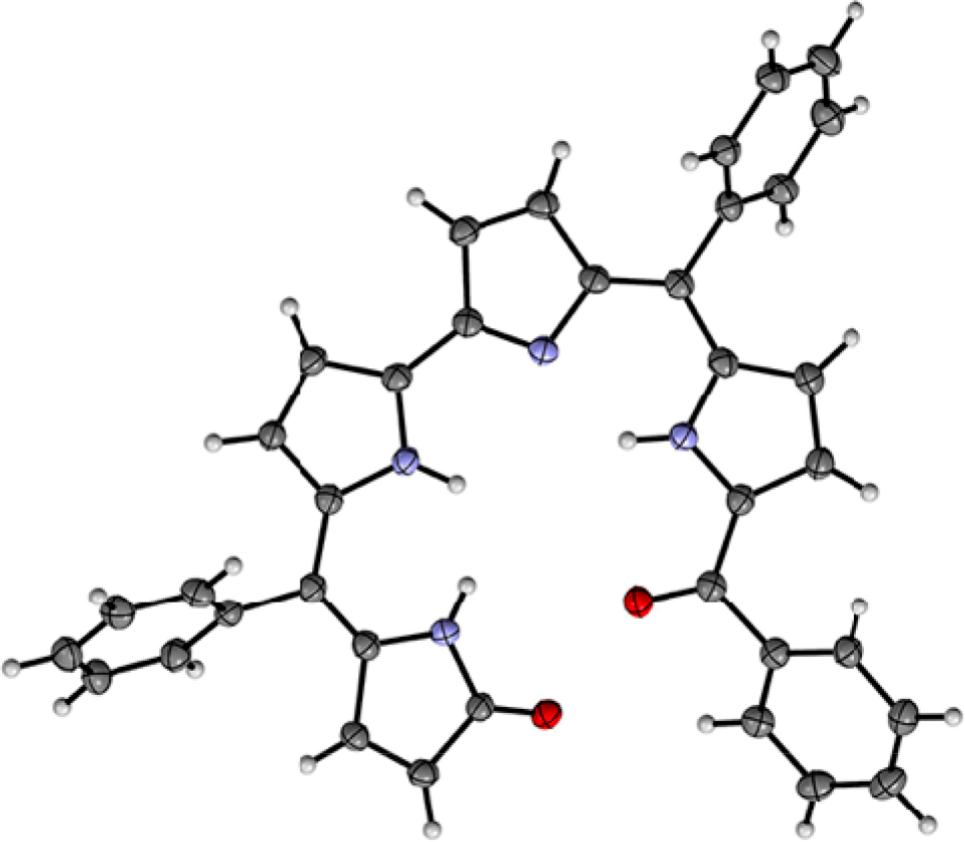
X-ray crystal structure of H_3_(OCTP) obtained from the reaction between H_3_(TPCorr) and zinc(II) salt as shown in Scheme 1.

The hypothesized pathway led us to explore the reaction of H_3_(TPCorr) with zinc(II) acetate dihydrate [Zn(OAc)_2_·2H_2_O] as shown in Scheme 1, where the progress of the reaction was monitored via UV–vis spectroscopy. Over the course of the reaction, spectral changes indicative of neutral corrole radical formation (*i.e.*, a slightly blue-shifted Soret band and the lack of well-defined Q-bands) were observed. However, the reaction workup afforded a product having a UV–vis spectrum characterized by a large broad absorption band centered at 553 nm (Figure 1) and a ^1^H NMR spectrum indicating the absence of an aromatic ring current (Figure S1). The X-ray crystal structure of the major product (Figure 2) confirmed the formation of an open-chain linear tetrapyrrole, [H_3_(OCTP)], thus suggesting that the oxidation reaction between H_3_(TPCorr) and Zn(OAc)_2_·2H_2_O under these solution conditions proceeded through a ring-opening reaction at the 10-position. A similar open-chain tetrapyrrole was identified in a report that studied the photodecomposition of meso-aryl substituted corrole under air.^19^

Attempts to synthesize the Ni(TPCorr) derivative utilizing Ni(OAc)_2_·4H_2_O under the same reaction conditions resulted in a similar outcome as outlined Scheme 1 where an oxidative degradation of the macrocycle was observed as opposed to the formation of a Ni(TPCorr) complex. When the limitation imposed by this synthetic protocol was considered, a new approach inspired by Bröring and co-workers’ reported procedure^18^ was elaborated. The new synthetic strategy consisted of *activating* the free base corrole macrocycle by first forming the neutral diprotic corrole radical H_2_(Corr^•^), which is more prone to coordinate divalent metals such as Zn^II^ and Ni^II^, followed by metalation with their respective acetate salts. As previously demonstrated,^20^ the oxidation of the free base corrole macrocycle utilizing iron(III) chloride effectively generates enough stable corrole radical needed for metalation of the divalent transition metal ions. Moreover, insertion of the zinc or nickel metal ions into the predisposed neutral corrole radical can be further promoted by performing the reaction in DMSO, a solvent that can potentially act as a labile neutral coordinating ligand as recently shown in the synthesis of new cobalt corrole derivatives.^21^

**Figure 3:**
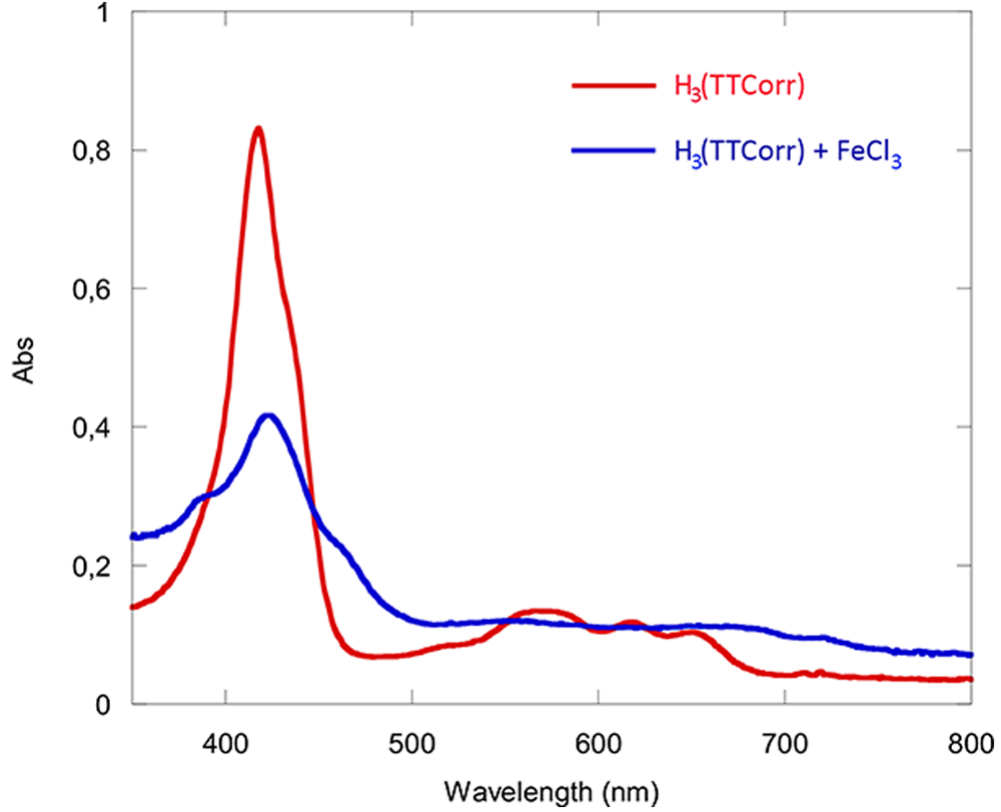
UV–visible spectrum of H_3_(TTCorr) in CH_2_Cl_2_ and the resulting spectrum observed 15 min after the addition of FeCl_3_ to the solution.

Following this revised scheme, the first set of experiments utilized a modified literature procedure in which 1 equiv. of FeCl_3_ was initially added to a DMSO solution containing H_3_(TTCorr) at 90 °C. The reaction progress was monitored by UV–vis spectroscopy, and after a few minutes, changes in the reaction mixture spectrum resulted in the disappearance of the Q-bands and a lower intensity Soret band consistent with the formation of a neutral diprotic corrole radical, H_2_(Corr^•^).^22^ The mixture was stirred for another 15 min until no further spectral changes were observed (Figure 3), after which 3 equiv. of Zn(OAc)_2_·2H_2_O was added to the solution.

**Figure 4:**
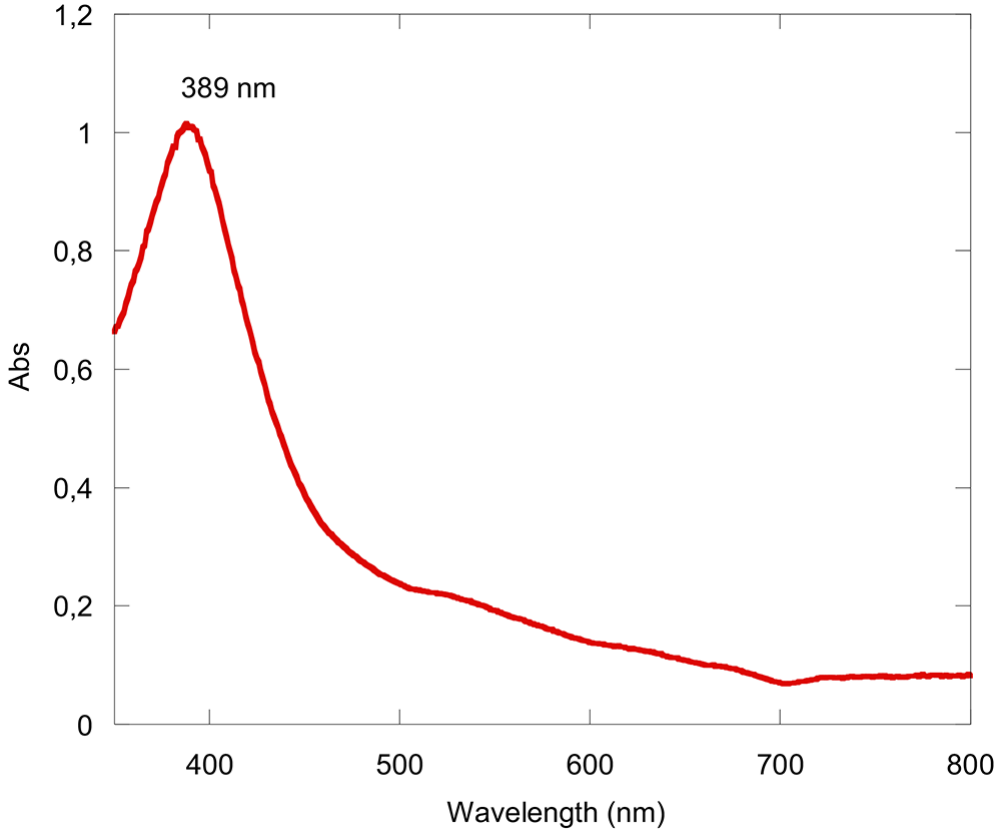
UV–visible spectrum of the crude reaction mixture obtained from the reaction of H_3_(TTCorr)/FeCl_3_/Zn(OAc)_2_·2H_2_O at 90 °C in CH_2_Cl_2_.

Upon addition of Zn(OAc)_2_·2H_2_O to the solution of chemically generated H_2_(TTCorr^•^), the color of the reaction mixture changed from green to brown, and after 40 min, the reaction was deemed complete as evidenced by the lack of further spectral changes. Brine was added to the resulting mixture, leading to the formation of a brown precipitate, which was filtered and dried. The solid was dissolved in CH_2_Cl_2_ and chromatographed over neutral alumina (10% deactivated) using neat CH_2_Cl_2_ as an eluent. The isolated fraction was then characterized according to its UV–vis and ^1^H NMR spectra. As shown in Figure 4, the UV–vis spectral pattern in CH_2_Cl_2_ displays a blue-shifted Soret-like band (λ_max_ = 389 nm) as compared to H_3_(TTCorr) with no other distinct characteristic bands (Figure 4).

Unexpectedly, ^1^H NMR analysis of the isolated fraction exhibited a signal pattern indicative of a diamagnetic molecule with the same molecular symmetry of H_3_(TTCorr). Thus, the UV–vis and ^1^H NMR spectroscopic data raised the concern that the isolated complex was actually the μ-oxo derivative O[Fe(TTCorr)]_2_. Washing the isolated fraction in CH_2_Cl_2_ with a 0.1 M HCl aqueous solution confirmed this postulate, resulting in a change of the UV–visible spectrum to the characteristic spectral pattern of an iron(III)chloride triarylcorrole complex.^23^ The formation of an iron corrole complex following this reaction protocol was not surprising, even if the syntheses of such complexes are normally carried out with an excess of iron(II) chloride. To gain a better understanding of the reaction mechanism, the experiment was repeated under the same solution conditions but without the addition of Zn(OAc)_2_·2H_2_O. Contrary to the expected synthesis and isolation of a Fe(TTCorr) complex, no iron corrole complex was observed and an analysis of the reaction mixture by UV–vis spectroscopy and TLC pointed toward H_2_(TTCorr^•^) as the main product. Further confirmation of the neutral diprotic H_2_(TTCorr^•^) radical was evidenced by the quantitative reduction of the resulting product upon addition of hydrazine, a mild reducing agent. The result of this control experiment led us to suspect that Zn^II^ played a role in the metalation of iron and the reaction moved toward three main steps: (1) formation of the neutral corrole radical, H_2_(TTCorr^•^); (2) insertion of the zinc ion to produce the zinc corrole complex, and (3) transmetalation of Fe(III) to the Zn(II) corrole affording the final Fe(TTCorr) complex, which was then converted to μ-oxo complex O[Fe(TTCorr)]_2_ in the presence of O_2_.

**Scheme 2:**
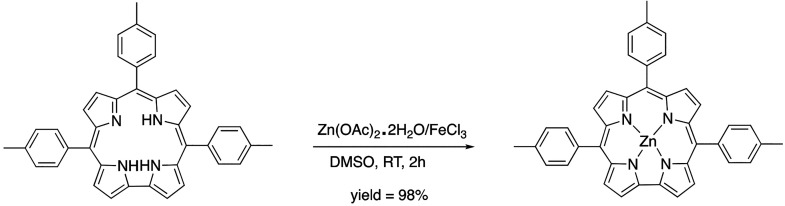
Synthetic Pathway for the Formation of Zn(TTCorr)

Since zinc triarylcorroles are suspected to be labile and only somewhat stable even at room temperature, we focused on attempting to lock-in the zinc coordination by performing the reaction at milder temperatures in the absence of light and under an inert atmosphere. Accordingly, H_3_(TTCorr) was dissolved in DMSO, and 1 equiv. of FeCl_3_ and 3 equiv. of Zn(OAc)_2_·2H_2_O were added (Scheme 2) while stirring the solution at room temperature until a quantitative reaction of the substrate was observed.

**Figure 5:**
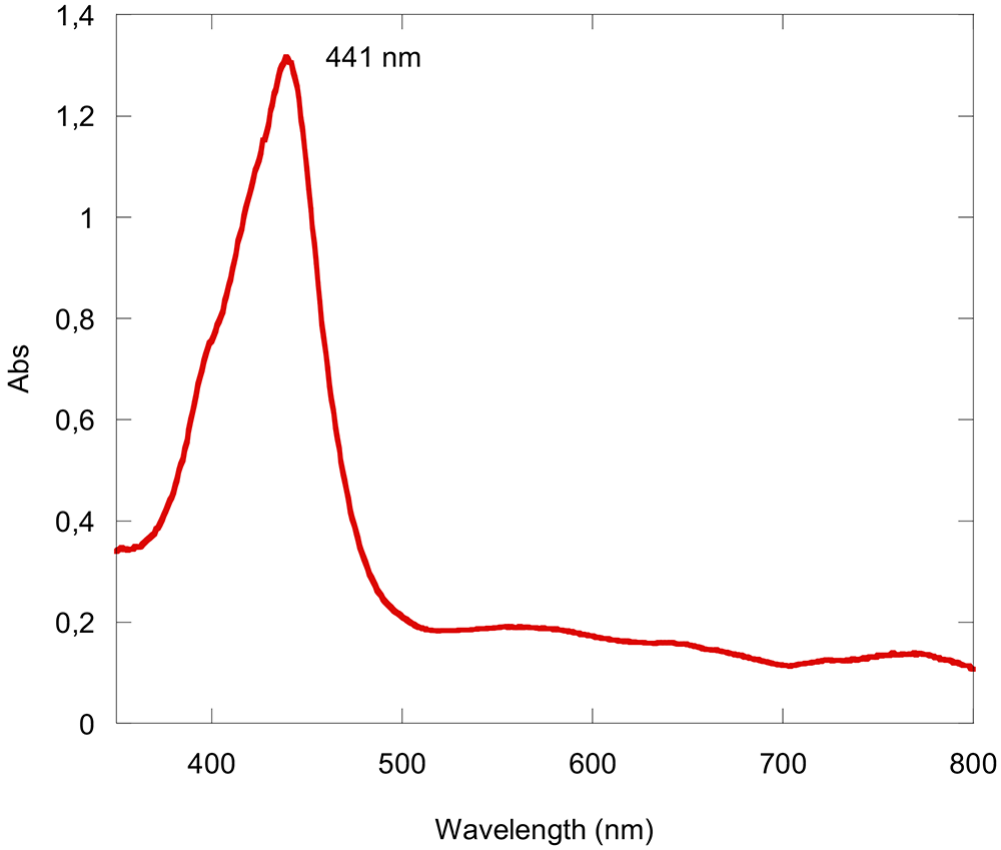
UV–visible spectrum of the crude reaction mixture obtained from the reaction of H_3_(TTCorr)/FeCl_3_/Zn(OAc)_2_·2H_2_O at room temperature in CH_2_Cl_2_.

Upon complexation of the Zn^II^ ion, the reaction mixture displayed a UV–vis spectrum in CH_2_Cl_2_ characterized by a broad red-shifted Soret band at 441 nm and broad ill-defined absorption bands of lesser intensity at lower energy (Figure 5), the latter of which is indicative for the expected Zn^II^(Corr^•^) character of the compound. The addition of brine to the crude reaction mixture resulted in a precipitate that was filtered and dried, affording a fine brown solid. Numerous attempts of chromatographic purification were then carried out to find the ideal conditions needed to isolate the desired compound.

Systematic purification experiments showed that (a) silica gel and neutral alumina together with chlorinated solvents, pure or as mixtures with MeOH/THF/toluene/EtOAc, quickly degrade the desired compound, leading to oxidized open-chain derivatives of the macrocycle and (b) basic alumina together with chlorinated solvents allowed for separation of only trace amounts of the desired compound. Optimal chromatographic conditions were obtained using basic alumina grade V and 5% CH_2_Cl_2_/pyridine (20:1, v/v) as an eluent. In this ideal chromatographic method, the coordination of a σ-donor axial ligand such as pyridine likely stabilizes the complex, preventing degradation as was observed for other metal corrole derivatives.^24^ The desired fraction was isolated in a 20% yield, and it was observed that further chromatographic purifications using the same conditions outlined above decreased the yield, leading to significant oxidative degradation of the compound. Chromatography on silica gel, a more acidic stationary phase, with 5% CH_2_Cl_2_/pyridine (20:1, v/v) led to a quantitative demetalation of Zn(TTCorr), regenerating the H_3_(TTCorr) starting material. This last observation supported the hypothesis that the main product was indeed the desired Zn(TTCorr) complex. Given the results of the various purification attempts outlined above, it is clear that Zn(TTCorr) is quite unstable for chromatography and the compound can be quantitatively obtained by direct precipitation from the crude reaction mixture. A similar result was also observed for the synthesis of cobalt triarylcorroles in DMSO. Thus, metalation of chemically generated H_2_(TTCorr^•^) with Zn^II^ following Scheme 2 was, once completed, quenched by adding brine, and the resulting solid was crystallized in CH_2_Cl_2_/hexane to provide the desired complex. Moreover, utilizing an alternative organic oxidant, such as 1 equiv. of *p*-chloranil, instead of iron(III) chloride, resulted in a final reaction mixture with spectroscopic data consistent with the formation of Zn(TTCorr); however, purification of the corrole product from the *p*-chloranil residue requires chromatographic procedures that are not compatible with the stability of the Zn(TTCorr) complex. For this reason, a mild inorganic oxidant such as FeCl_3_ remained the best choice due its solubility in aqueous media, making it easily separated from the hydrophobic Zn(TTCorr) complex. It is also worth mentioning that, when insertion of the zinc ion was complete after a 2 h reaction at room temperature, heating the crude reaction mixture led to the formation of the iron metallocorrole as described above. Nonetheless, Zn(TTCorr) crystallized from CH_2_Cl_2_/hexane was fully characterized by spectroscopic and electrochemical methods as described on the following pages.

Given the rather reactive nature of the isolated Zn(TTCorr) and literature reports suggesting that the presence of electron-withdrawing chlorine substituents in the 3,17 positions of corrole strongly improved the stability of the corrole radical, we decided to study the zinc insertion into the 3,17-dibromo-5,10,15-tritolylcorrole H_3_(3,17-Br_2_TTCorr), symmetrically functionalized on the β-positions with two bromine atoms, to observe if this approach can be generalized to increase the stability of the final Zn complex.

**Scheme 3:**
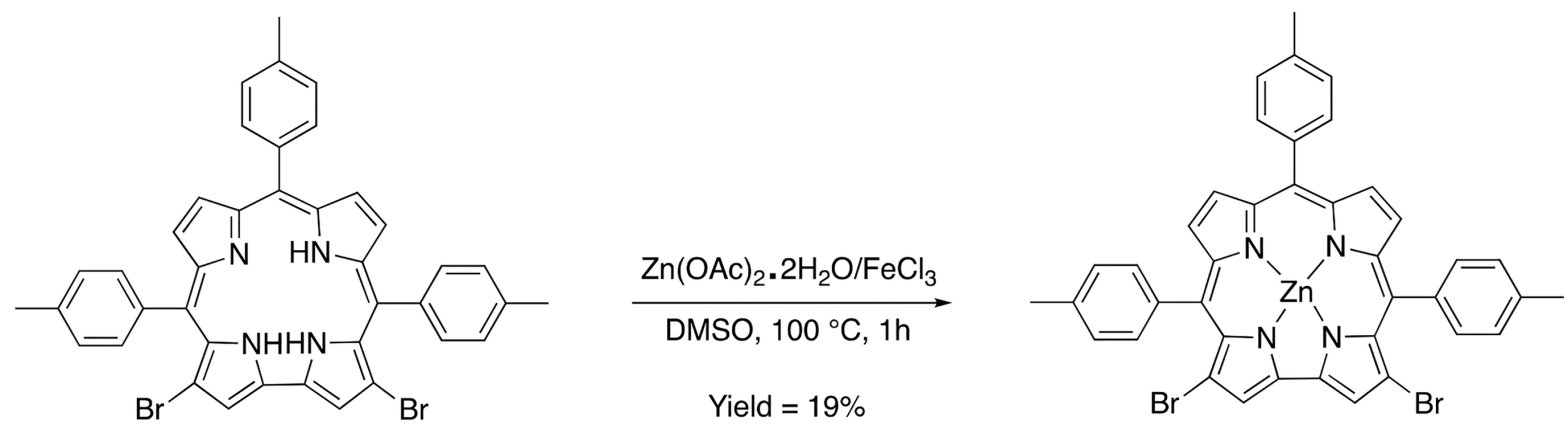
Synthetic Pathway for the Formation of the Zinc Corrole Complex Zn(3,17-Br_2_TTCorr)

The procedure was performed with the same reaction conditions adopted for H_3_(TTCorr) (Scheme 3), but in this case, the synthesis did not lead to a quantitative formation of the complex, leaving some unreacted starting material. Increasing the quantity of FeCl_3_/Zn(OAc)_2_·2H_2_O or extending the reaction time did not significantly improve the yield of the complex, and for this reason, a chromatographic purification procedure was required to separate the desired product from the starting material.

**Figure 6:**
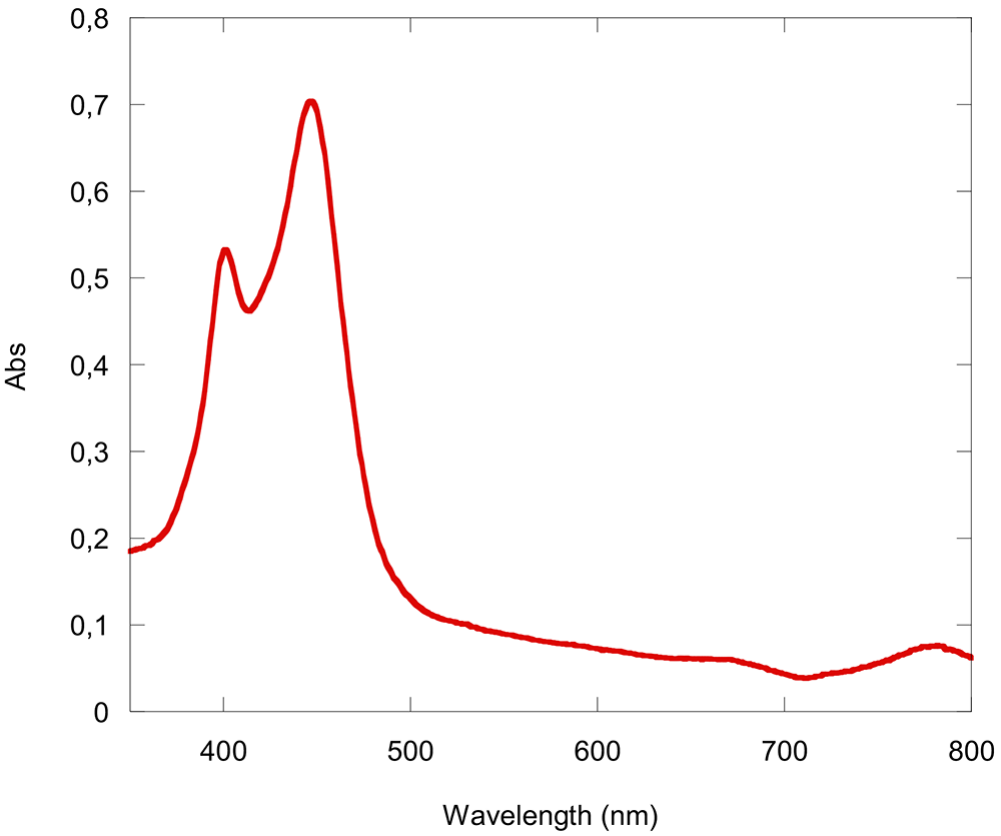
UV–visible spectrum of Zn(3,17-Br_2_TTCorr) obtained from the reaction of H_3_(3,17-Br_2_TTCorr)/FeCl_3_/Zn(OAc)_2_·2H_2_O at room temperature in CH_2_Cl_2_.

While the electron-withdrawing nature of the bromine atoms makes the oxidation more difficult and consequently the formation of the corresponding active neutral radical species [H_2_(3,17-Br_2_TTCorr^•^)] is able to react with Zn^II^ ions, the substitution is beneficial for the stability of the final Zn(3,17-Br_2_TTCorr) product as evidenced by its ability to undergo chromatographic separation (Figure 6).

^1^H NMR analysis of Zn(TTCorr) highlighted the paramagnetic property of this complex, which was confirmed and further investigated by electron paramagnetic resonance (EPR) spectroscopy.

**Figure 7:**
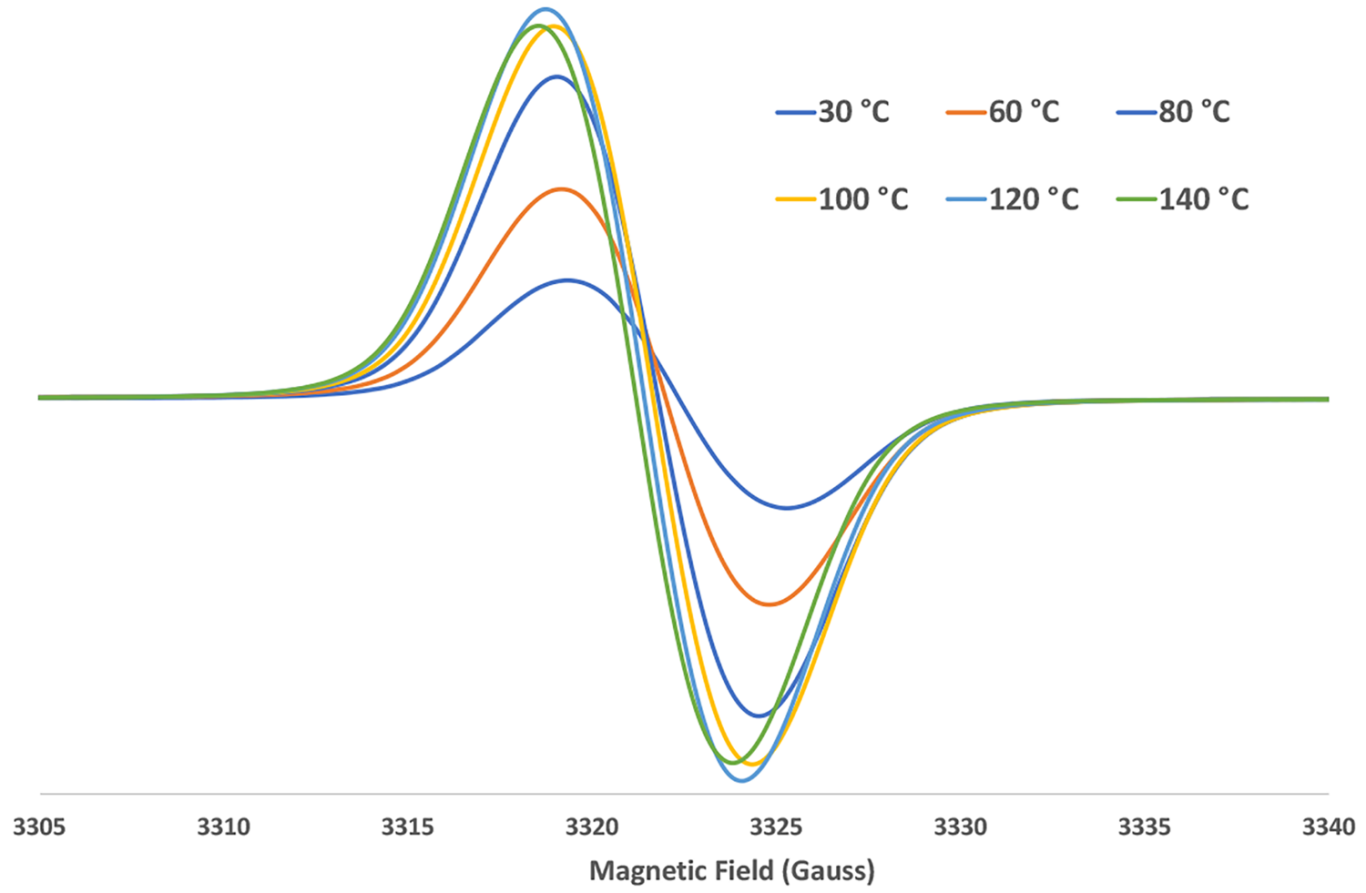
Isotropic EPR spectra of Zn(TTCorr) in a *tert*-butyl benzene deaerated solution at different temperatures.

**Figure 8:**
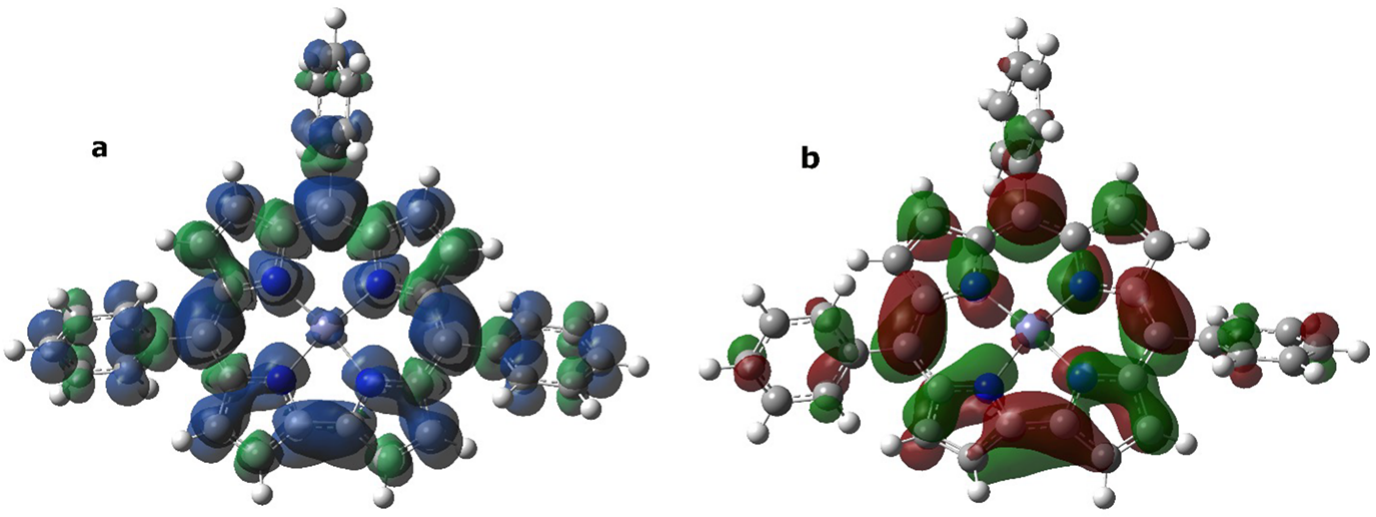
Zn(TTCorr) (α–β) spin density distribution (a) and singly occupied molecular orbital (SOMO) (b) plots computed at the PBE0/6-31G(d) level. Positive spin densities are shown in blue (a) and red (b), while negative ones are in green.

The EPR spectrum of Zn(TTCorr) in *tert*-butyl benzene is characterized by a broad signal centered at *g* = 2.0026, a value very close to that of the free electron, *g* = 2.0023, and is reported in Figure 7 at different temperatures. However, it is worth pointing out that in general the isotropic *g*-factor of organic free radicals deviates from the corresponding value of the free electron due to spin–orbit coupling effects arising from the contribution of each individual atom in the molecule; as a consequence, the contribution of a specific atom (or group of atoms) will be larger as the odd electron is more delocalized onto that atom (or group of atoms), producing a corresponding deviation of *g* from the free electron value.^25^ Since a value of *g* = 2.0032 has been recorded for the simple H_2_(3,17-Cl_2_Corr^•^),^18^ the corresponding value recorded for Zn(TTCorr) outlines the influence exerted by the Zn^II^ ion in such a complex. In order to gain a deeper insight into the experimental EPR data, density functional theory (DFT) calculations were carried out starting from the optimized geometries of both free base radical, H_2_(TTCorr^•^), and Zn(TTCorr). The data obtained indicate the effect of the metal in the Zn(TTCorr) complex on the unpaired electron distribution. In fact, its computed spin density plot in Figure 8a shows that, in line with the results obtained for H_2_(TTCorr^•^), the unpaired electron is mainly delocalized on the corrole moiety, but a small positive density on the metal has been found; the corresponding singly occupied molecular orbital (SOMO) plot, depicted in Figure 8b, reveals the marked p character of this orbital.

Moreover, the DFT computed molecular geometry for Zn(TTCorr) is also very close to that of the neutral H_2_(TTCorr^•^). In particular, the plane identified by the four “pyrrole” nitrogen atoms showed a small 4° distortion in the H_2_(TTCorr^•^) radical, while a distortion of 17° has instead been found in Zn(TTCorr), likely due to the presence of a square planar coordinated Zn(II) ion; moreover, the N–Zn distances are about 1.94 Å, and the distance between two opposite nitrogen atoms of the “corrole cavity” is 3.85 Å in Zn(TTCorr), while it is 3.71 Å in the H_2_(TTCorr^•^) neutral radical. Finally, when the temperature of a Zn(TTCorr) deaerated solution was stepwise increased to 140 °C directly in the EPR cavity, the signal was shifted toward lower magnetic fields, and the final signal was centered at *g* = 2.0033, a value identical with that found for the H_2_(TTCorr^•^) radical, as shown in Figure 7, thus suggesting a likely elimination of the metal from the complex.

The *m*/*z* value obtained from the TOF-SIMS MS analysis of the molecule (Figure S3) confirmed the formation of Zn(TTCorr). The complex was stable in the solid state for a few weeks at room temperature under N_2_ atmosphere, but in solution, it decomposes and prevents any attempts to obtain crystals suitable for X-ray characterization. Zn(3,17-Br_2_TTCorr) instead shows better stability in solution than Zn(TTCorr), similar to what was observed by Bröring and co-workers with dichloro-β-substituted aryl corrole.^18^

#### Electrochemistry and Spectroelectrochemistry of Zinc Corroles

**Figure 9:**
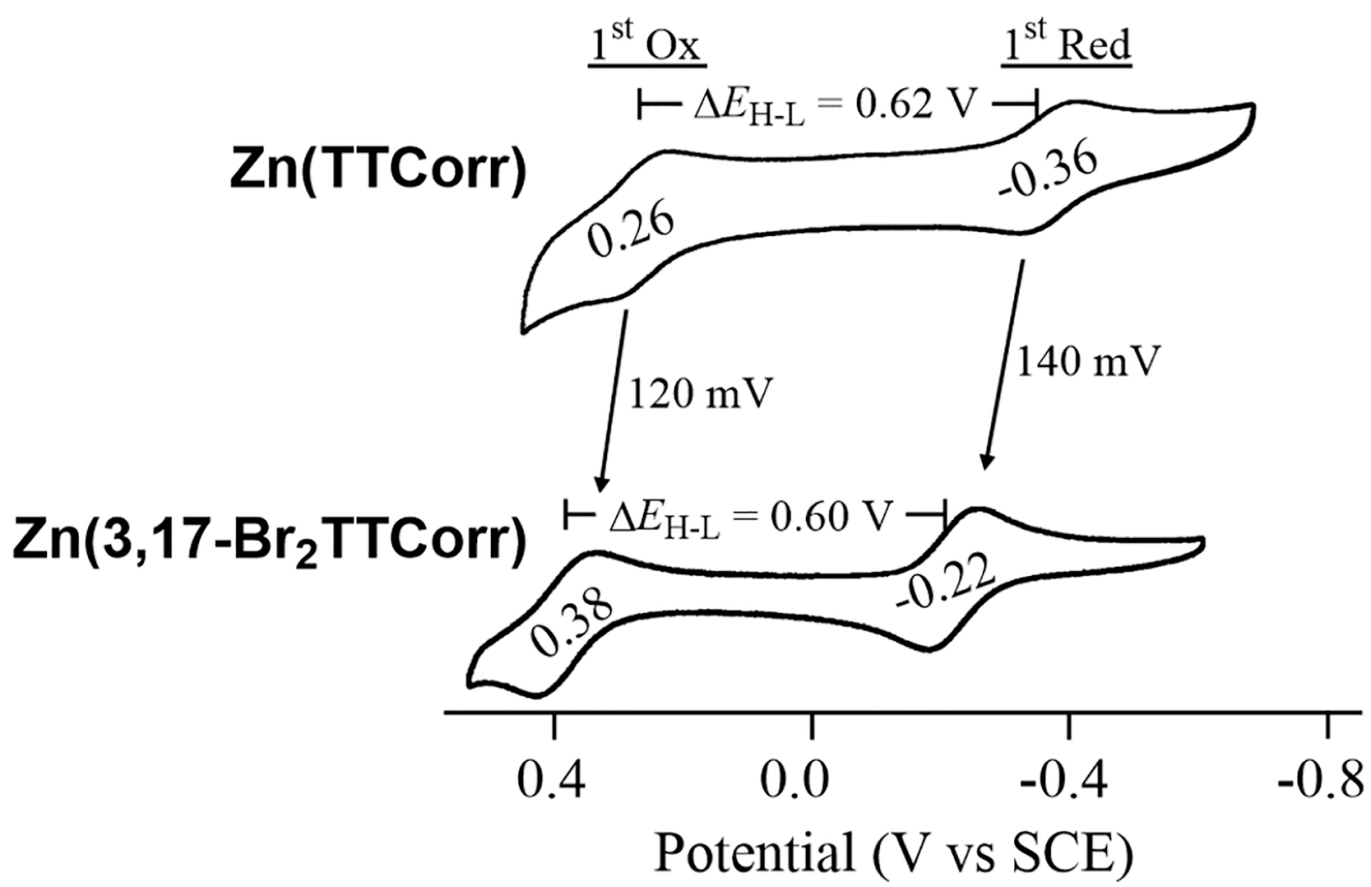
Cyclic voltammograms of Zn(TTCorr) and Zn(3,17-Br_2_TTCorr) in pyridine/CH_2_Cl_2_ (1:45, v:v) containing 0.1 M TBAP. Scan rate = 0.1 V/s.

The electrochemical behaviors of Zn(TTCorr) and Zn(3,17-Br_2_TTCorr) were examined in pyridine/CH_2_Cl_2_ (1:45, v:v) containing 0.1 M TBAP, and the spectral properties of the singly reduced and singly oxidized species under these solution conditions were investigated by thin-layer spectroelectrochemistry. Cyclic voltammograms comparing the first reversible reduction and first reversible oxidation of the two zinc(II) corrole radicals are shown in Figure 9, and the potentials along with their electrochemically measured HOMO–LUMO gaps are summarized in Table 1. As seen in the figure, both complexes exhibit reversible first reduction and first oxidation processes where the ratio of the cathodic current (*i*_pc_) over the anodic current (*i*_pa_) is 1.00, indicating that the product formed upon electron addition or abstraction is stable under these solution conditions. The first reduction of Zn(TTCorr) is facile (*E*_1/2_ = −0.36 V vs SCE) and is shifted anodically by 140 mV upon going to Zn(3,17-Br_2_TTCorr) (*E*_1/2_ = −0.22 V). The easier reduction for the dibromo derivative as compared to Zn(TTCorr) is consistent with an inductive effect stemming from the addition of two electron-withdrawing β-Br substituents on the periphery of the corrole macrocycle. Moreover, the shift in the first reduction amounts to 70 mV per β-Br substituent, a nearly identical value to that observed for various metalloporphyrins were the stepwise addition of bromo groups to the β-pyrrole positions of the macrocycle (from mono- to octabrominated porphyrins) has been shown to shift the first reduction by 60 mV/Br, independent of the central metal ion.^26−31^

**Table 1:** Half-Wave Potentials (V vs SCE) for the Two Investigated Zinc Corroles in Pyridine/CH_2_Cl_2_ (1:45, v:v) Containing 0.1 M TBAPF_6_

	*E*_1/2_, V vs SCE		
cpd	1st ox	1st red	Δ*E*_H-L_
Zn(TTCorr)	0.26	–0.36	0.62
Zn(3,17-Br_2_TTCorr)	0.38	–0.22	0.60

A similar analysis can be made for the first oxidation potential of the two investigated zinc corroles. As seen in Figure 9, the first electron abstraction of Zn(TTCorr) occurs at *E*_1/2_ = 0.26 V vs SCE while the same process for Zn(3,17-Br_2_TTCorr) (at *E*_1/2_ = 0.38 V) is 120 mV more positive due to the electron-withdrawing bromo substituents at the 3,17-positions, amounting to a 60 mV shift per β-Br substituent. The nearly identical anodic shift in the first oxidation and first reduction results in essentially identical HOMO–LUMO gaps (Δ*E*_H-L_) for the two zinc(II) corroles of 0.62 and 0.60 V for Zn(TTCorr) and Zn(3,17-Br_2_TTCorr), respectively (see Table 1).

These Δ*E*_H-L_ values for the Zn(II) corrole radicals are notably smaller than other non-innocent corroles with electroinactive metal centers such as copper corroles, which typically exhibit Δ*E*_H-L_ values of 0.90 ± 0.07 V.^9,32−38^ On the other hand, the HOMO–LUMO gap of structurally analogous nickel corrole was shown to exist as a dimer in solution and exhibit Δ*E*_H-L_ ∼ 0.70 V when measured in CH_2_Cl_2_/0.1 M TBAP.^10^

**Figure 10:**
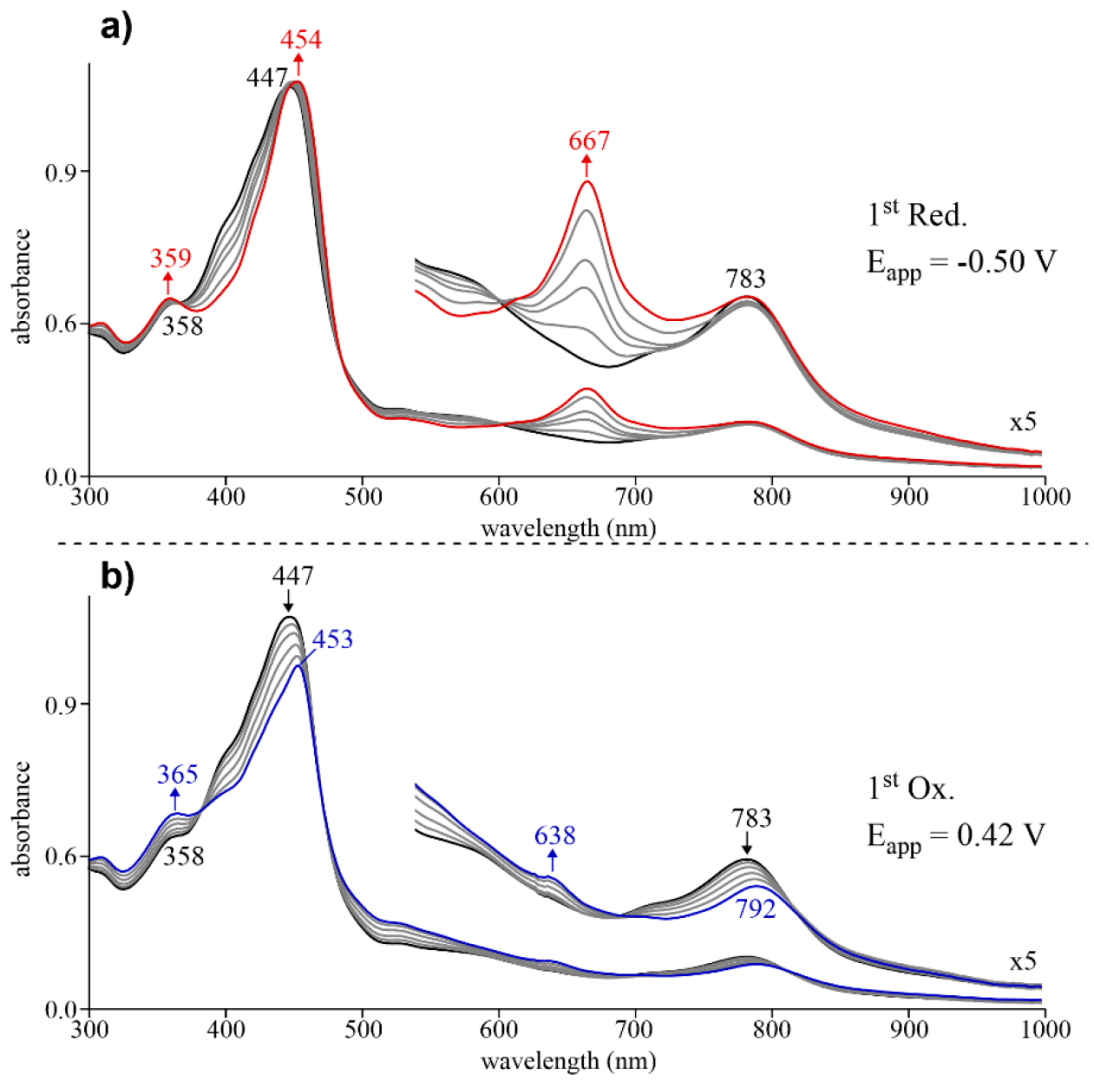
Thin-layer spectral changes associated with (a) the first reduction and (b) the first oxidation of Zn(TTCorr) in pyridine/CH_2_Cl_2_ (1:45, v:v) containing 0.1 M TBAP.

**Figure 11:**
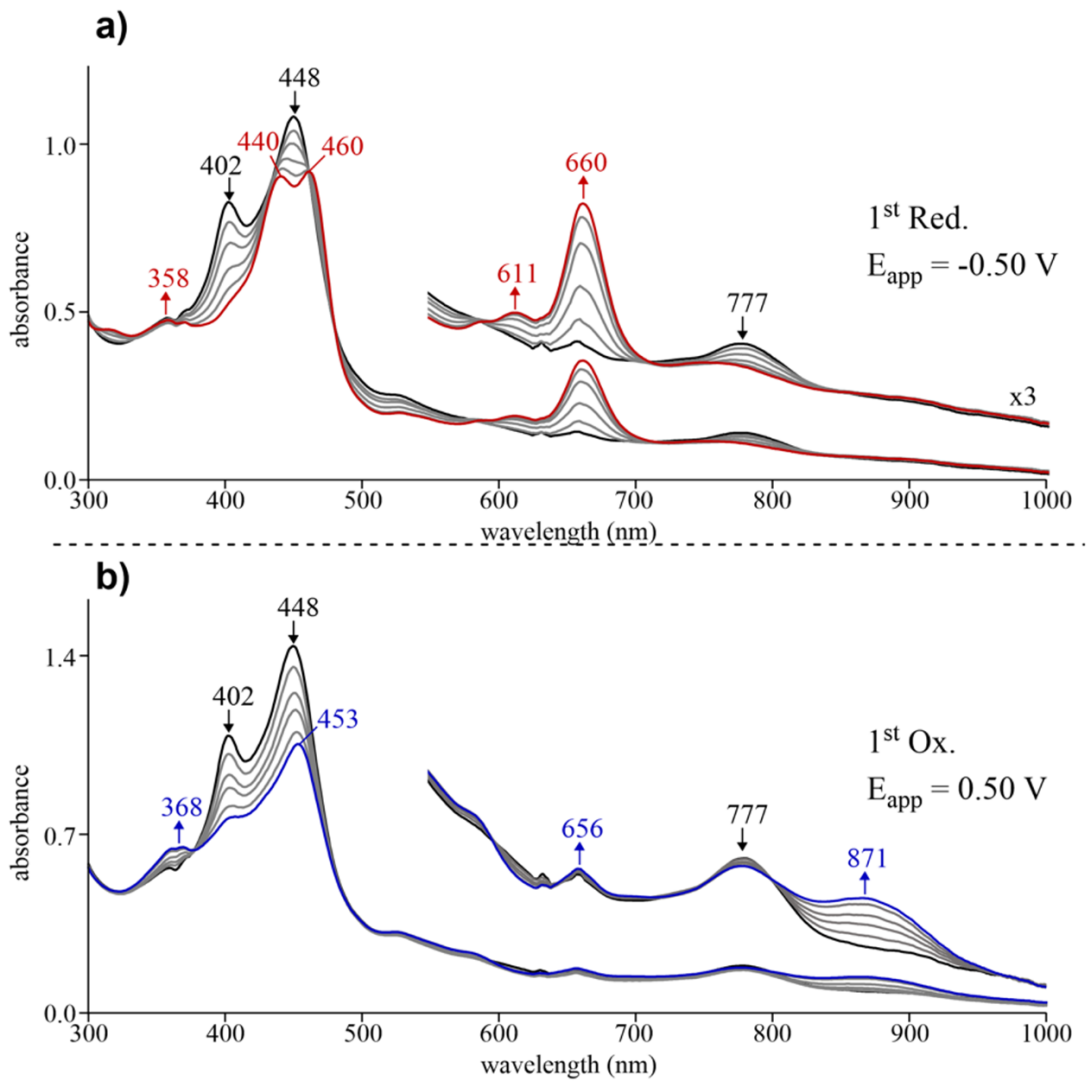
Thin-layer spectral changes associated with (a) the first reduction and (b) the first oxidation of Zn(3,17-Br_2_TTCorr) in pyridine/CH_2_Cl_2_ (1:45, v:v) containing 0.1 M TBAP.

In order to further characterize these zinc corrole systems, the evolution of the one-electron reduced and one-electron oxidized species was monitored by thin-layer spectroelectrochemistry in pyridine/CH_2_Cl_2_ (1:45, v:v) containing 0.1 M TBAP. Prior to analysis of the spectral changes shown for Zn(TTCorr) (Figure 10) and Zn(3,17-Br_2_TTCorr) (Figure 11), it is worth noting that divalent zinc is, for all intents and purposes, an electroinactive metal ion, and thus, the addition or abstraction of electrons from these systems is expected to strictly occur at the corrole macrocycle. Indeed, this expectation is borne out by the spectroelectrochemical results detailed in the discussion below.

As seen in Figure 10a, the initial spectrum of the neutral Zn(II) corrole radical prior to application of a reduction potential has a Soret band at 447 nm, a characteristic radical band at 783 nm, and no well-defined Q-bands due to disruption of the π-system. It is worth noting that this spectrum, especially the low-energy band (at 783 nm) ascribed to the corrole radical character, is strikingly similar to that of a previously reported Zn(II) dichloro-trimesitylcorrole radical.^18^ Upon application of a controlled reducing potential, the Soret band is red-shifted to 454 nm with the appearance of a new well-defined Q-band at 667 nm. This observation, particularly the appearance of a well-defined Q-band, is an expected result for the addition of an electron to the corrole macrocycle, which strengthens the π-system of the initial Zn(II) corrole radical as given by eq 1:
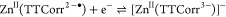
1

It is worth noting that the spectrum of the singly reduced species, [Zn^II^(TTCorr^3–^)]^−^, is strikingly similar to other M(II) corrole^3–^ systems such as singly reduced copper^28−31,33−36^ or nickel^36^ corroles, all of which display a red-shifted Soret band and at least one well-defined Q-band as compared to their non-innocent, neutral M^II^(TTCorr^2–•^) counterparts. Moreover, non-innocent cobalt corroles also display similar spectral changes upon reduction (*i.e.*, a red-shifted Soret band and the appearance of a well-defined Q-band upon the one-electron addition to Co^II^(Corr^2–•^)).^21,39−41^

Figure 10b displays the spectral changes observed upon the one-electron oxidation of Zn(TTCorr). In this the case, the Soret band of the neutral Zn(II) corrole radical at 447 nm decreases in intensity along with a concomitant decrease of the radical band at 783 nm. The final spectra of the singly oxidized species exhibit a Soret band at 453 nm of lesser intensity and two weak, low energy absorptions at 638 and 792 nm. The decreased Soret band of the singly oxidized complex is blue-shifted as compared to the neutral complex, similar to the spectral changes observed during the oxidation of non-innocent copper triarylcorroles.^28,33^ On the basis of this result, along with the highly unfavorable oxidation of the Zn(II) central metal ion, the one-electron oxidation is assigned to occur at the corrole macrocycle giving a cation as shown in eq 2:
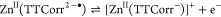
2

Similar to the spectral changes seen upon reduction of Zn(TTCorr), the one-electron addition to Zn(3,17-Br_2_TTCorr) (Figure 11a) results in a red-shift of the “split” Soret bands from 402 and 448 nm in the neutral complex to 440 and 460 nm in the singly reduced species along with a concomitant appearance of a well-defined Q-band at 660 nm. As described above, the appearance of this new Q-band for [Zn(3,17-Br_2_TTCorr^3–^)]^−^ is indicative of an electron addition to the macrocycle, which restores the corrole π-system from the initial neutral radical in solution prior to electron transfer. The one-electron oxidation of Zn(3,17-Br_2_TTCorr) (Figure 11b) also results in quite similar spectral changes as those seen for the nonbrominated derivative (Figure 10b) in that a decreased intensity of the major Soret band at 448 nm is observed and shifted to 453 nm while two weak absorptions appear in the visible region at 656 and 871 nm. Again, the decreased Soret band of singly oxidized Zn(3,17-Br_2_TTCorr) is blue-shifted as compared to the neutral complex, similar to the spectral changes observed during the oxidation of other non-innocent triarylcorroles (*vide supra*).^9,28−30,33−40^

### Synthesis and Characterization of Nickel Corroles

Experimental data gathered from the Zn(TTCorr) complex synthesis and isolation were adapted to set up a working protocol for metalation with nickel. Initial attempts using H_3_(TPCorr)/H_3_(TTCorr) and nickel(II) acetate tetrahydrate in chlorinated solvents and methanol led again to oxidative degradation of the macrocycle.

**Scheme 4:**
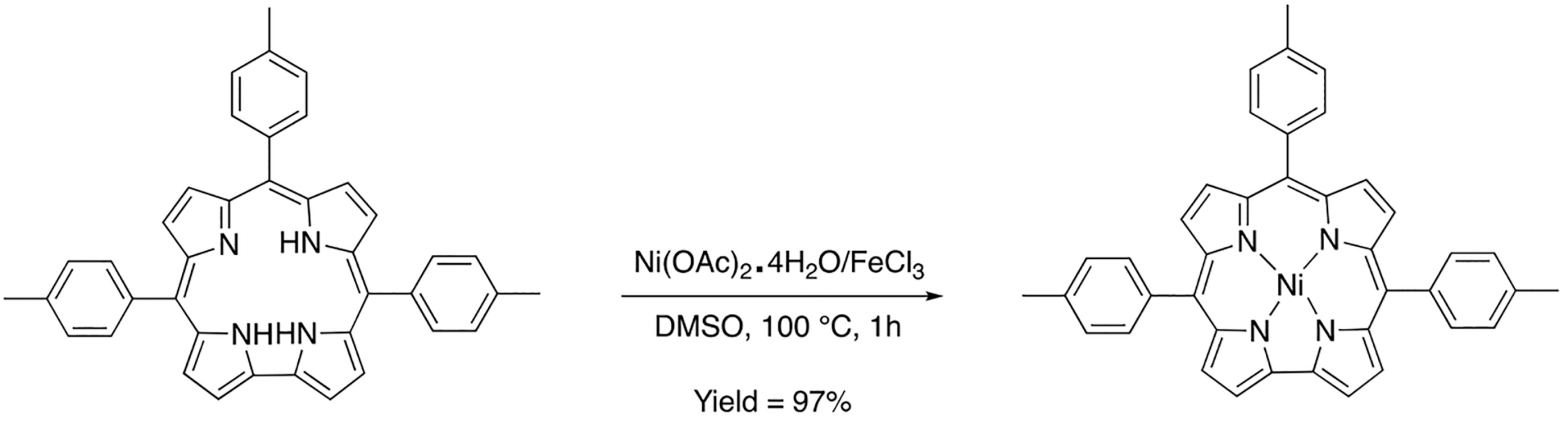
Synthetic Pathway for the Formation of Ni(TTCorr)

**Figure 12:**
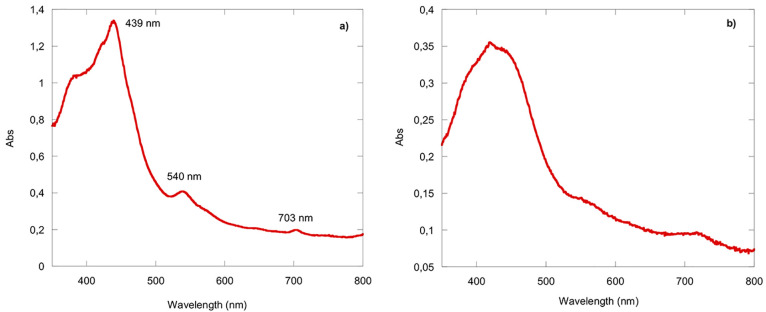
(a) Reaction mixture after 1 h and (b) isolated Ni(TTCorr) product.

For this reason, the solvotropic properties of DMSO proved to be successful for nickel insertion. To obtain a quantitative reaction of H_3_(TTCorr) with nickel, it was necessary to increase the reaction temperature to 100 °C for 1 h (Scheme 4). Longer reaction times at room temperature resulted in lower yields with slow degradation of the starting material and Ni(TTCorr). The desired product was isolated by precipitation with brine, dried, and crystallized in CH_2_Cl_2_/hexane. Indeed, similar to what was observed for Zn(TTCorr), chromatographic attempts to isolate Ni(TTCorr) decreased the yield substantially. The UV–visible spectrum of the crude reaction mixture (after 1 h) in CH_2_Cl_2_ and the crystallized product are shown in Figure 12a,b, respectively.

The two spectra in Figure 12a,b differ significantly in shape. For example, the spectrum of Ni(TTCorr) in the reaction mixture is characterized by sharp peaks at 439, 540, and 703 nm, but when the compound is crystallized, these signals become broader. This phenomenon can possibly be attributed to the role of DMSO, a solvent that can potentially act as a weak axial electron donor ligand coordinating to the nickel center and is present in the crude reaction mixture but is lost after the work up.^8^

**Figure 13:**
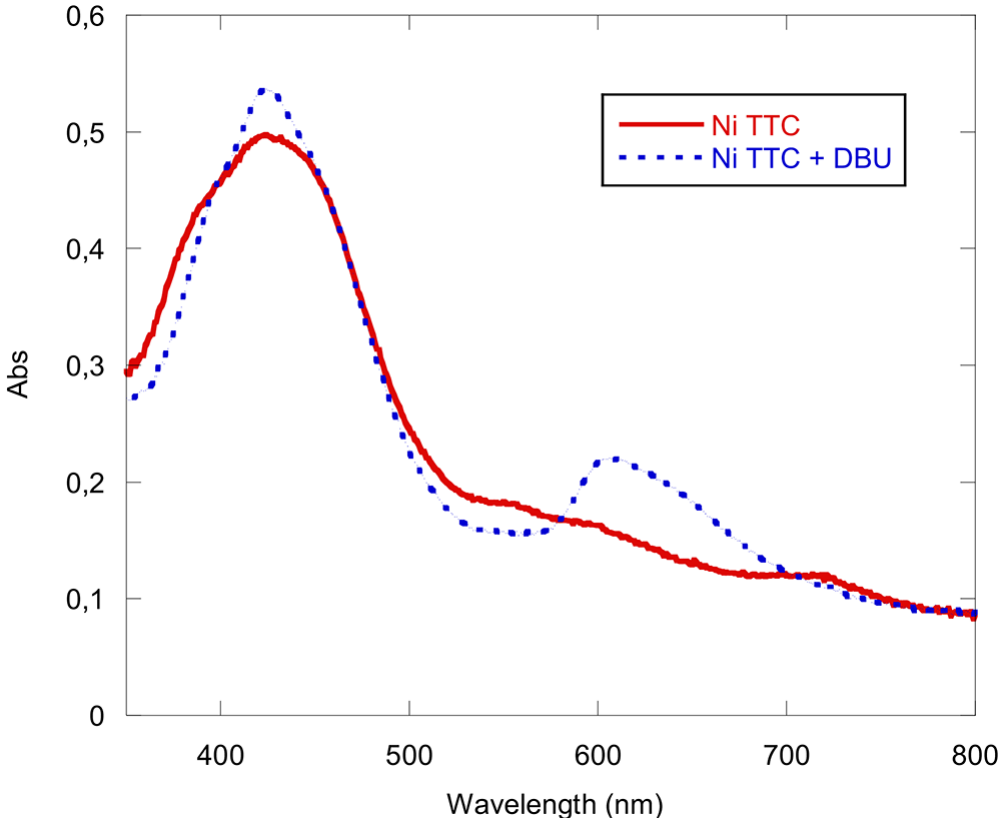
Ni(TTCorr) (solid line), Ni(TTCorr) + 5 equiv. of DBU (dotted line) in CH_2_Cl_2_.

Ni(TTCorr) in solution can undergo a reductive reaction when titrated with [1,5-diazabiciclo(5.4.0)undec-7-ene] (DBU) (Figure 13). This mild reducing agent changes the optical properties of the complex and enhances its aromatic character as suggested by a sharper Soret band at 425 nm and the appearance of a well-defined Q-band at 605 nm, an identical spectrum to that observed for electroreduced Ni(TTCorr) (Figure S6).^10^ Moreover, it is also worth noting that the same spectrum can be generated via the addition of cyanide anions (Figure S7), Lewis basic anions were previously shown to reduce non-innocent copper corroles via anion induced electron transfer.^9^ Together, these results indicate the non-innocent nature of the isolated nickel complex with an electronic structure best represented as Ni^II^(TTCorr^•^).

Mass spectrometry analysis of the precipitate by TOF-SIMS MS confirmed a successful synthesis of the Ni(TTCorr) complex (Figure S4).

The paramagnetic nature of Ni(TTCorr) is also evident by ^1^H NMR and EPR spectroscopy. As described below, the behavior of the Ni(TTCorr) complex is somewhat different than that of its zinc analogue.

**Figure 14:**
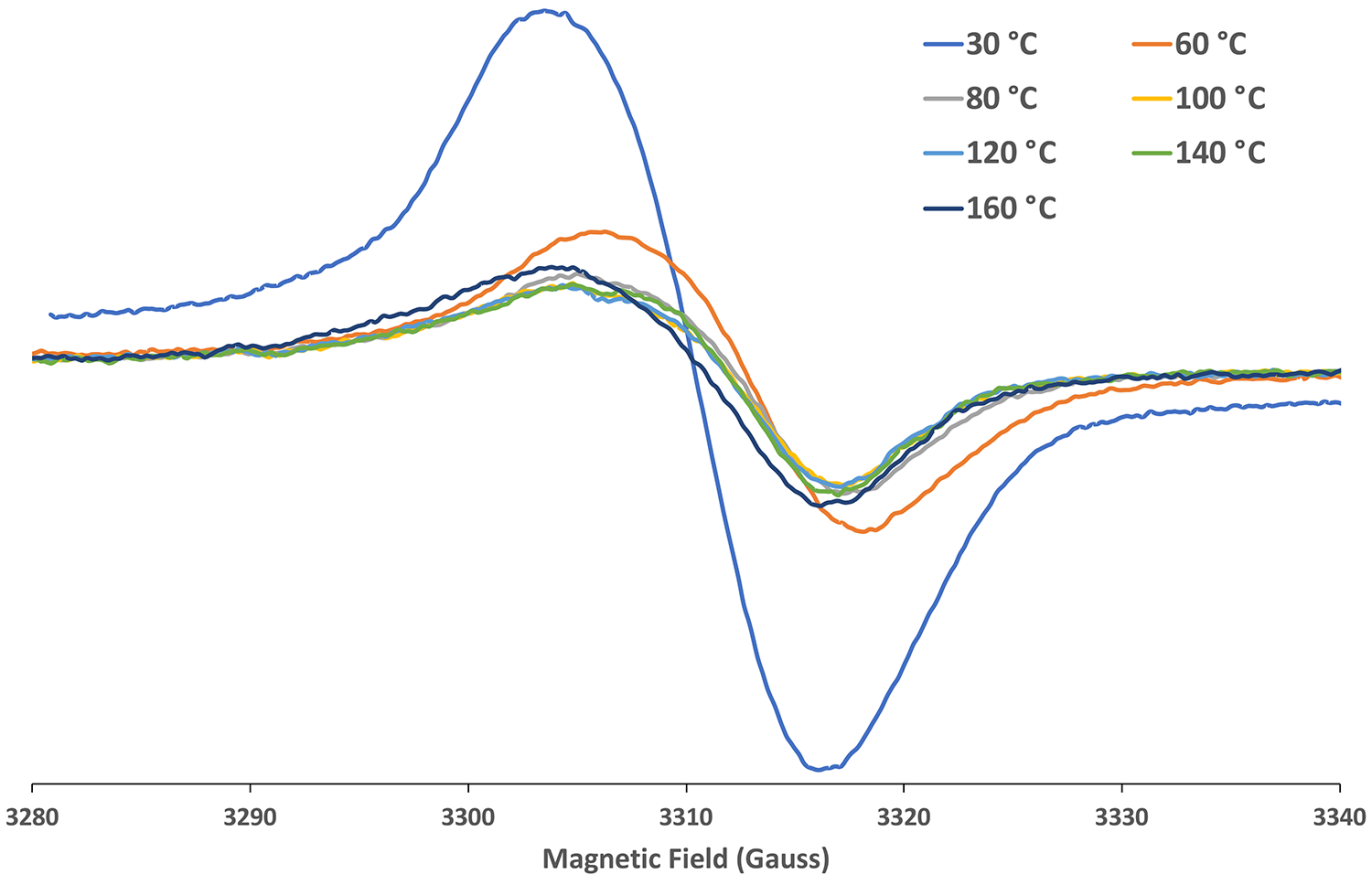
Isotropic EPR spectra of Ni(TTCorr) in a *tert*-butyl benzene deaerated solution at different temperatures.

In the case of Ni(TTCorr), the EPR spectrum (Figure 14) is always represented by a broad signal but centered at *g* = 2.0017, a significantly different value with respect to that of the free electron, indicating the marked effect exerted by the metal in this compound. When the temperature was increased, as was done for Zn(TTCorr), the EPR signal was shifted toward higher magnetic fields, but in this case, the residual signal at 160 °C was still centered at *g* = 2.0033, the same value found for the H_2_(TTCorr^•^) radical, indicating an elimination of the metal.

**Figure 15:**
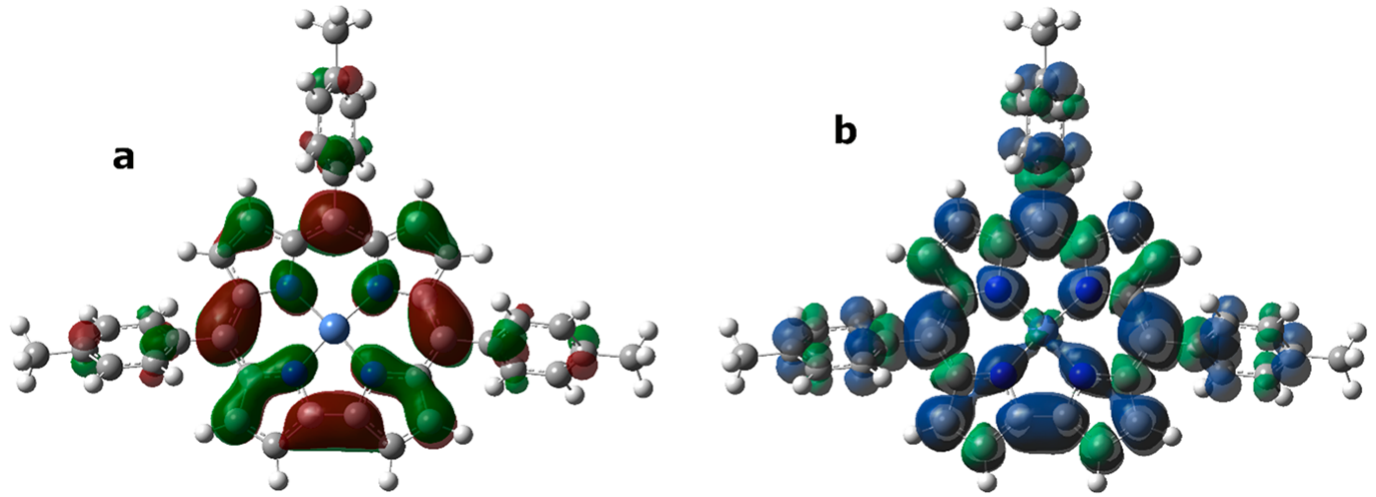
Ni(TTCorr) singly occupied molecular orbital (SOMO) (a) and spin density distribution (b) plots computed at the PBE0/6-31G(d) level. Positive spin densities are shown in red (a) and blue (b), while negative ones are in green.

Again, in an attempt to gain a better understanding of EPR results, DFT analysis was performed (Figure 15) to further probe the electronic structure of Ni(TTCorr). Figure 15a shows the corresponding SOMO plot and reveals a marked p character, but unlike the zinc derivative, the metal is not directly involved. Moreover, the computed spin density distribution plot in Figure 15b shows that the radical is mainly delocalized on the corrole moiety, as was found for the Zn^II^ analogue; however, a negative spin density is instead present on the metal.

Concerning the optimized molecular geometry, the N–Ni distances are about 1.85 Å, and the plane identified by the four pyrrole nitrogen atoms showed a very small distortion of 1.5°. These findings are in line with the presence of square planar Ni^II^, a smaller ion with respect to Zn^II^ (63 vs 74 pm ionic radius, respectively). In addition, the distance between two opposite nitrogen atoms in the corrole cavity is 3.70 Å, very close to the value of 3.71 Å found for H_2_(TTCorr^•^).

## Conclusions

Ni(II) and Zn(II) complexes, which are among the most common porphyrin derivatives, are rarely reported in the case of corroles. We have circumvented this problem in the case of the Zn complex through a preliminary formation of the H_2_(TTCorr^•^) neutral radical corrole by oxidation with FeCl_3_ in DMSO. The consequent reaction with Zn acetate in the same solvent allowed the formation of the Zn(TTCorr) derivative. The obtained complex is sufficiently stable to allow its EPR and spectroelectrochemical characterization, which confirmed the radical character of the macrocycle.

Zn(TTCorr) significantly decomposes in solution by ring opening to form an open-chain tetrapyrrole, and the same species was formed if the Zn insertion was attempted directly with H_3_(TTCorr). Moreover, when Zn insertion into the H_2_(TTCorr^•^) radical was attempted at higher temperatures, transmetalation occurred and the corresponding iron complex was obtained. EPR studies confirmed demetalation of the Zn complex by increasing the temperature, which can then lead to Fe complexation. However, the insertion of two bromine atoms as substituents at the 3,17 positions strongly stabilized the resulting complex, allowing a better characterization of the desired Zn derivative. This approach appears to be a promising way for the preparation of stable Zn corrole complexes.

The use of DMSO also allows for the coordination of a Ni(II) ion in the corrole ring, again with the need for oxidation of the initial free base corrole to H_2_(TTCorr^•^). The resulting complex can be simply obtained by precipitation from the solution with the addition of water, thus improving the reaction yield because chromatographic purification induced some decomposition of the complex.

EPR also confirmed the radical character of the Ni(II) complex. Demetalation also occurred in this case when increasing the temperature, confirming the limited stability of the complex. In both cases, the use of DMSO as reaction solvent allows for a simple separation of the reaction product by precipitation with water, avoiding the need for chromatographic separation that significantly reduces the reaction yield.

## Experimental Section

### Materials and Methods

Reagents and solvents (Aldrich) were of the highest grade available and were used without further purification. Thin-layer chromatography (TLC) was performed on Sigma-Aldrich silica gel plates. Chromatographic purification of the reaction products was accomplished using silica gel 60 (70–230 mesh, Sigma–Aldrich) as stationary phase. UV–vis spectra were measured on a Varian Cary 50 Spectrophotometer using CH_2_Cl_2_ as solvent. NMR experiments were performed in deuterated acetone at 15 °C and recorded with a Bruker Avance spectrometer operating at 400 MHz for ^1^H. The EPR measurements were carried out by the research group of Prof. Pierluigi Stipa at the Polytechnic University of Marche. Mass spectrometry (MS) spectra (TOF-SIMS) were recorded using a positive method with a TOF-SIMS V (IONTOF) spectrometer or with a MALDI-TOF Bruker Autoflex II using α-cyano-4-hydroxycinnamic acid as the matrix at the University of Stockholm.

Isotropic X-band EPR spectra were recorded on a Bruker EMX/Xenon spectrometer system equipped with a variable temperature apparatus, a microwave frequency counter, and an NMR Gauss meter for field calibration; for *g*-factor determination, the whole system was standardized with a sample of perylene radical cation in concentrated sulfuric acid (*g*-factor = 2.00258).

Density functional theory calculations^42^ were carried out using the Gaussian 09 suite of programs^43^ at Cineca Supercomputing Center.^44^ All calculations were performed at the PBE0/6-31G(d) level using a proper basis set for Zn and Ni atoms.^45^ Geometry optimizations were carried out with the unrestricted formalism, giving ⟨*S*^2^⟩ = 0.7507 ± 0.0003 for spin contamination (after annihilation). Isotropic *g*-factors were determined by means of the gauge independent atomic orbital method as the averaged value of the *xx*, *yy*, and *zz* corresponding components.

Electrochemical measurements were performed at 298 K using an EG&G Princeton Applied Research (PAR) Model 173 potentiostat/galvanostat paired with an EG&G PAR Model 175 universal programmer and a Houston Instruments Omnigraphic 2000 XY Plotter. The three-electrode system used for cyclic voltammetric measurements consisted of a glassy carbon working electrode, a platinum counter electrode, and a saturated calomel reference electrode (SCE), which was separated from the bulk of the solution by a fritted glass bridge of low porosity. The bridge was purchased from Gamry Instruments and contained the solvent/supporting electrolyte mixture.

Thin-layer UV–vis spectroelectrochemical measurements were performed with a home-built cell containing a light transparent platinum net-working electrode, a platinum wire counter electrode, and an SCE reference electrode. Potentials were applied and monitored with an EG&G PAR Model 173 potentiostat. High purity argon gas was used to deoxygenate the solution prior to and during each spectroelectrochemical experiment.

### X-ray Crystallography

The experimental and refinement details for H_3_(OCTP) are given below. Single-crystal X-ray data were measured using a dual-source Rigaku SuperNova diffractometer equipped with an Atlas detector and an Oxford Cryostream cooling system using mirror-monochromated Cu Kα radiation (λ = 1.54184 Å). Data collection and reduction was performed using the program *CrysAlisPro*,^46^ and a Gaussian face-index absorption correction method was applied.^47^ The structure was solved with direct methods (*SHELXT*)^48^ and refined by full-matrix least-squares based on *F*^*2*^ using *SHELXL*-2015.^48^ All non-hydrogen atoms were refined anisotropic displacement parameters. Hydrogen atoms were placed in idealized positions and included as riding atoms. Isotropic displacement parameters for all H atoms were constrained to multiples of the equivalent displacement parameters of their parent atoms with U_iso_(H) = 1.2U_eq_ (parent atom). The X-ray single crystal data and experimental details as well as CCDC numbers are given below.

### H_3_(OCTP)

5,10,15-Triphenylcorrole (20 mg, 0.38 mmol) and Zn(II) acetate (40 mg, 2.6 mmol) were dissolved in CHCl_3_ (20 mL), and the solution was refluxed for 1 h under air. The solvent was evaporated under vacuum, and the residue was purified by column chromatography (alumina gel, CHCl_3_ as eluent) to give the corresponding open-chain product (3.8 mg, 18%). Elem. Anal. Calcd for C_37_H_26_N_4_O_2_: C, 79.55; H, 4.69; N, 10.03%. Found: C, 79.13; H, 4.64; N, 9.98%.

UV–vis (CH_2_Cl_2_), λ_max_ [nm] (ε, L mol^–1^ cm^–1^): 395 (3.84 × 10^4^), 553 (2.71 × 10^4^). MS (MALDI-TOF): *m*/*z* calcd. for C_37_H_26_N_4_O_2_ 558.21 found 558.024. ^1^H NMR (400 MHz, CDCl_3_) δ 8.30 (br, 3H) 7.96 (d, *J* = 7.2 Hz, 2H), 7.64 (t, *J* = 7.4 Hz, 1H), 7.60–7.38 (m, 11H), 7.09–6.99 (m, 3H), 6.98 (d, *J* = 4.7 Hz, 1H), 6.83 (d, *J* = 3.9 Hz, 1H), 6.68 (d, *J* = 3.6 Hz, 1H), 6.25 (d, *J* = 5.6 Hz, 1H), 1.44–1.26 (m, 3H), 0.99–0.86 (m, 4H).

Crystal data for H_3_(OCTP) (obtained via CHCl_3_/hexane): CCDC-2194501, C_37_H_26_N_4_O_2_, brown plate, 0.19 × 0.17 × 0.05 mm^3^, triclinic, space group *P*1̅ (No. 2), *a* = 10.0249(3) Å, *b* = 10.8859(3) Å, *c* = 13.8627(4) Å, α = 71.940(1)°, β = 86.966(2)°, γ = 77.643(1)°, *V* = 1404.84(7) Å^3^, *Z* = 2, *D*_calc_ = 1.321 gcm^–3^, *F*(000) = 584, μ = 0.661 mm^–1^, *T* = 120(2) K, θ_max_ = 72.49°, 22 685 total reflections, 4888 with *I*_o_ > 2σ(*I*_o_), *R*_int_ = 0.0290, 5543 data, 397 parameters, 4 restraints, GooF = 1.049, *R*_1_ = 0.0337 and w*R*_2_ = 0.0843 [*I*_o_ > 2σ(*I*_o_)], *R*_1_ = 0.0390 and w*R*_2_ = 0.0884 (all reflections), −0.203 < Δρ < 0.224 eÅ^–3^

### 5,10,15-Tritolylcorrolato Zn(II) Zn(TTCorr)

5,10,15-Tritolylcorrole (46 mg, 0.08 mmol) was dissolved in DMSO (15 mL), and FeCl_3_ (13 mg, 0.08 mmol) and Zn(II) acetate dihydrate (53 mg, 0.24 mmol) were added to the solution. The reaction mixture was stirred at room temperature and monitored by UV–vis spectroscopy. After 2 h, brine was added and the reaction product was precipitated, filtered, washed several times with pure water, and dried. The brown powder was dissolved in CH_2_Cl_2_ and crystallized in CH_2_Cl_2_/hexane 1:3 v/v to afford Zn(TTCorr) as a brown product (49 mg, 98%). Elem. Anal. Calcd for C_40_H_29_N_4_Zn: C, 76.13; H, 4.63; N, 8.88%. Found: C, 76.03; H, 4.60; N, 9.08%. UV–vis (CH_2_Cl_2_), λ_max_ [nm] (ε, L mol^–1^ cm^–1^): 441 (9.12 × 10^4^). (TOF-SIMS) *m*/*z* calcd. for C_40_H_29_N_4_Zn 629.1684, found 629.1685.

### 3,17-Dibromo-5,10,15-tritolylcorrole H_3_(3,17-Br_2_TTCorr)

This derivative was synthesized according to the procedure reported in the literature.^49^

### 3,17-Dibromo-5,10,15-tritolylcorrolato Zn(II) Zn(3,17-Br_2_TTCorr)

3,17-Dibromo-5,10,15-tritolylcorrole (10 mg, 0.014 mmol) was dissolved in DMSO (5 mL), and FeCl_3_ (2.3 mg, 0.014 mmol) and zinc(II) acetate dihydrate (9.2 mg, 0.042 mmol) were added to the solution. The reaction mixture was stirred at room temperature, and the progress of the reaction was monitored by UV–vis spectroscopy. After 12 h, brine was added and the product was filtered, washed several times with water, and dried. The dark powder was dissolved in a minimum amount of CH_2_Cl_2_–pyridine (20:1 v/v) and chromatographed on basic alumina grade V. The first brown fraction was collected and dried to give 2.1 mg (yield 19%) of the desired compound. UV–vis (CH_2_Cl_2_), λ_max_ [nm] (ε, L mol^–1^ cm^–1^): 400 (6.23 × 10^4^), 446 (8.51 × 10^4^).

### [5,10,15-Tritolylcorrolato Ni(II)] [Ni(TTCorr)]

5,10,15-Tritolylcorrole (50 mg, 0.09 mol) was dissolved in DMSO (15 mL), and FeCl_3_ (14 mg, 0.09 mmol) and Ni(II) acetate tetrahydrate (46 mg, 0.27 mmol) were added. The solution was refluxed for 60 min and cooled to room temperature, and brine was added to the mixture; then, the product was precipitated, filtered, washed several times with pure water, and dried. The brown powder was dissolved in CH_2_Cl_2_ and crystallized in CH_2_Cl_2_/hexane 1:3 v/v to afford Ni(TTCorr) as a brown product (52.8 mg, 97%). Elem. Anal. Calcd for C_40_H_29_N_4_Ni: C, 76.94; H, 4.68; N, 8.97%. Found: C, 76.90; H, 4.62; N, 9.01%. UV–vis (CH_2_Cl_2_), λ_max_ [nm] (ε L mol^–1^ cm^–1^): 436 (3.01 × 10^4^), 707(4.71 × 10^3^). (TOF-SIMS) *m*/*z* calcd. for C_40_H_29_N_4_Ni 623.1746, found 623.1724.

## Supporting Information Available

^1^H NMR, MALDI-TOF, and spectroelectrochemical characterization of compounds (PDF)
