# Supplementary material for: The Difficult Marriage of Triarylcorroles with Zinc and Nickel Ions
Source: Inorg Chem. 2022 Oct 26;61(44):17790–803. doi: 10.1021/acs.inorgchem.2c03099 (PMC9644369; doi:10.1021/acs.inorgchem.2c03099)
Supplement: Supplementary file 1 — ic2c03099_si_001.pdf [file ic2c03099_si_001.pdf]

Supporting Information

The Difficult Marriage of Triarylcorroles with Zinc and Nickel ions

Mario L. Naitana,[a,†] W. Ryan Osterloh,[b] Lorena Di Zazzo,[a] Sara Nardis,[a] Fabrizio Caroleo,[a] Pierluigi Stipa,[c] Khai-Nghi Truong,[d] Kari Rissanen,[d] Yuanyuan Fang,[b] Karl M. Kadish,[b] and Roberto Paolesse[a]**

AUTHOR ADDRESS

a) Department of Chemical Science and Technologies, University of Rome Tor Vergata. Via della Ricerca Scientifica, 00133 Roma, Italy. E-mail: roberto.paolesse@uniroma2.it

b) Department of Chemistry, University of Houston, Houston, TX, USA. E-mail: kkadish@uh.edu

c) Dipartimento di Scienze e Ingegneria della Materia, dell'Ambiente ed Urbanistica, Università Politecnica delle Marche, Via Brecce Bianche 12, 60131 Ancona, Italy

d) Department of Chemistry, University of Jyväskylä, 40014 Jyväskylä, FINLAND

† Present address: Department of Science, Roma Tre University, Via della Vasca Navale 84, 00146 Rome, Italy

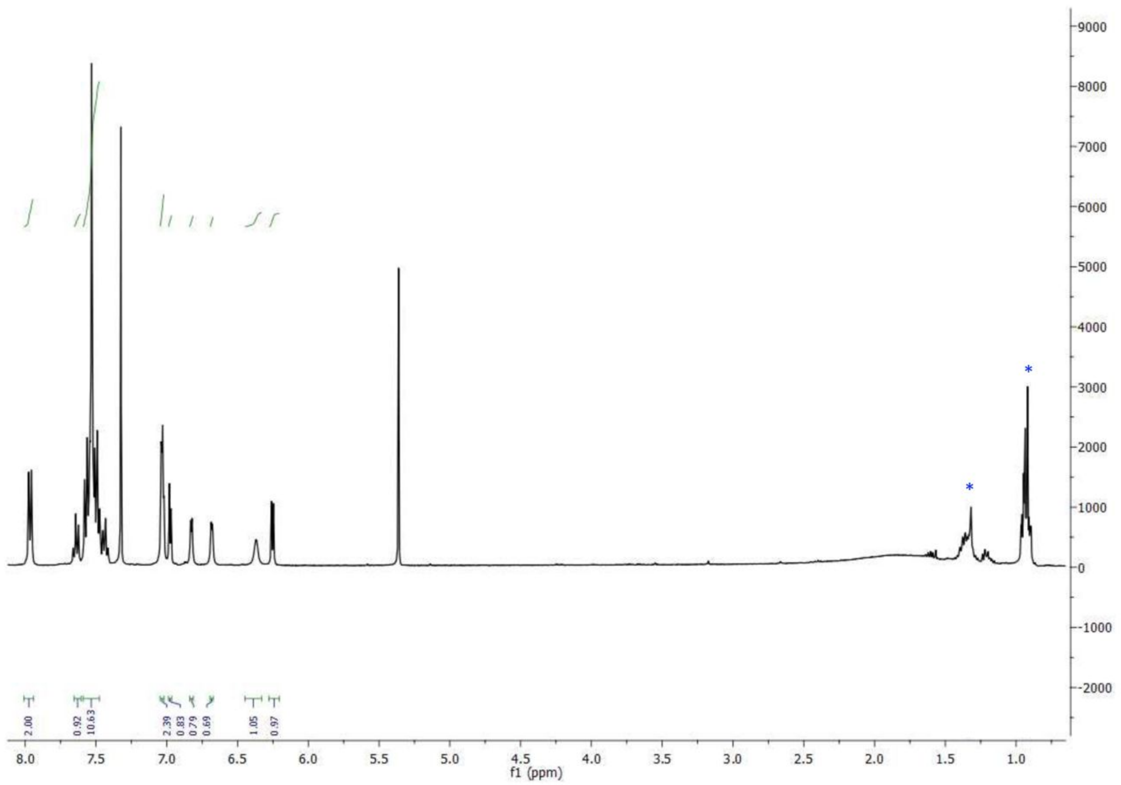

Figure S1. ^{1}H-NMR spectrum of **H_3(OCTP)** in $CDCl_3$ (400 MHz, *298K)*.

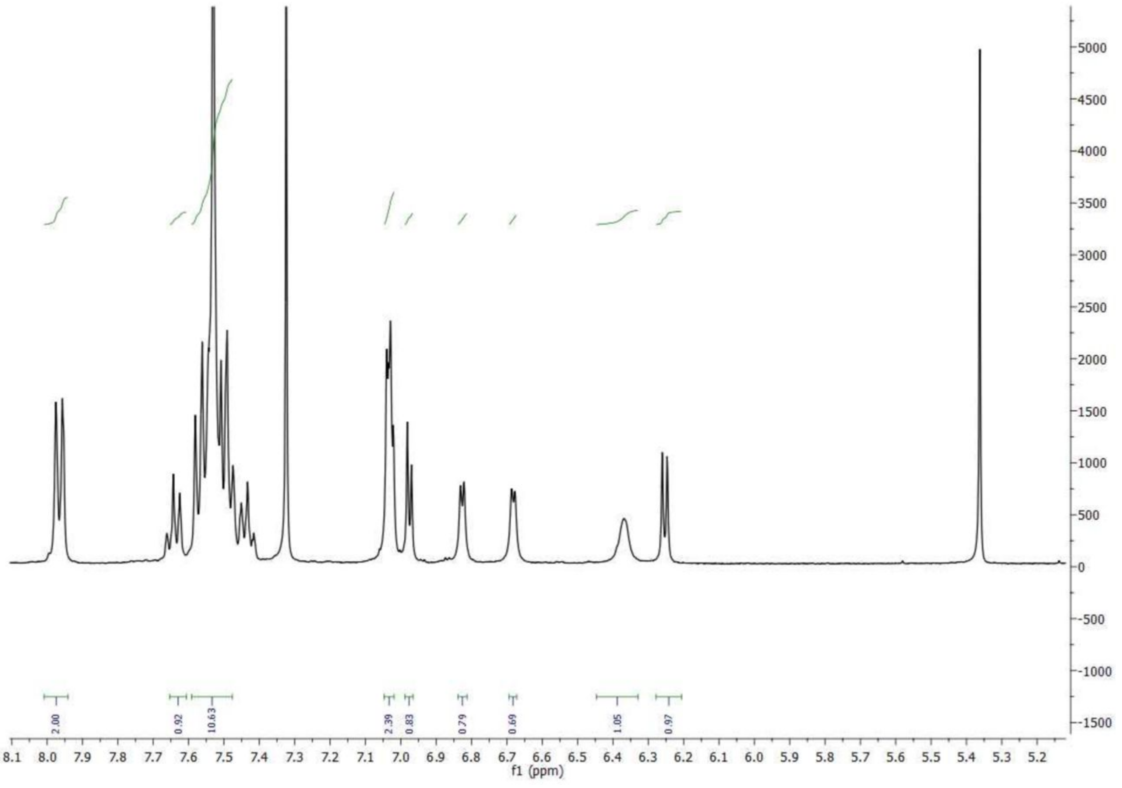

Figure S2. Aromatic region of the ^{1}H-NMR spectrum of **H_3(OCTP)** in $CDCl_3$ (400 MHz, *298K)*.

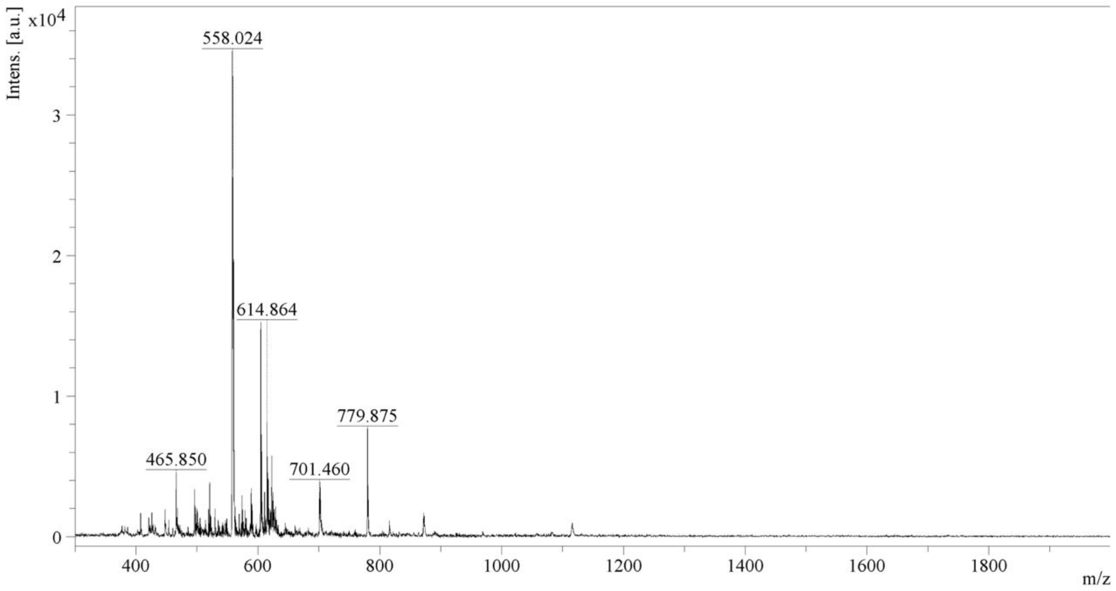

Figure S2. MALDI-TOF spectrum of $\mathbf{H_3(OCTP)}$

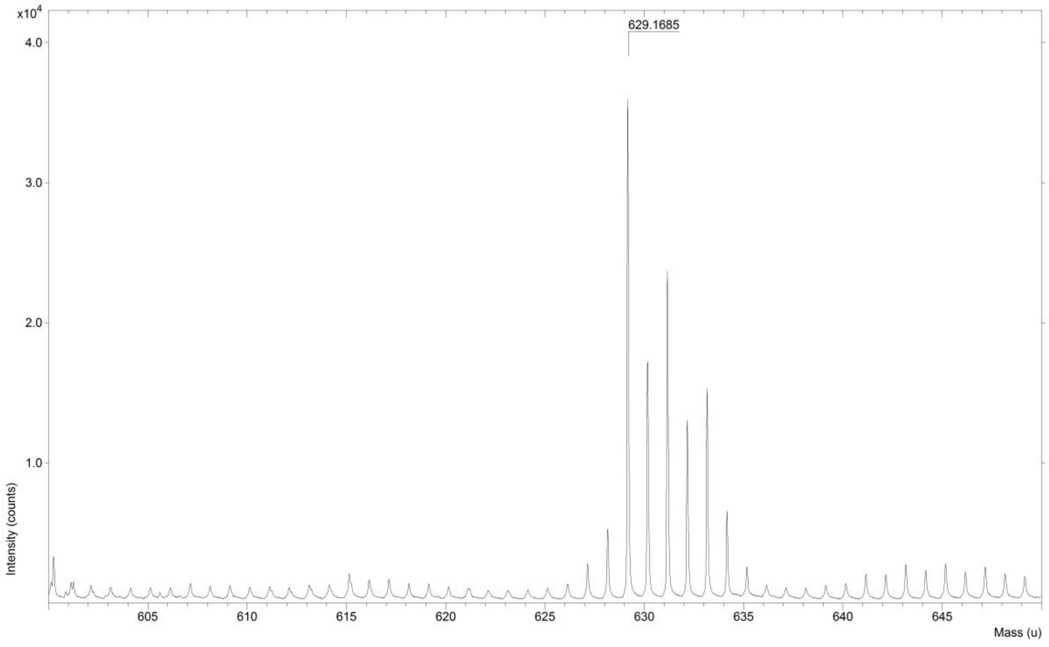

Figure S3. TOF-SIMS spectrum of **Zn(TTCorr)**.

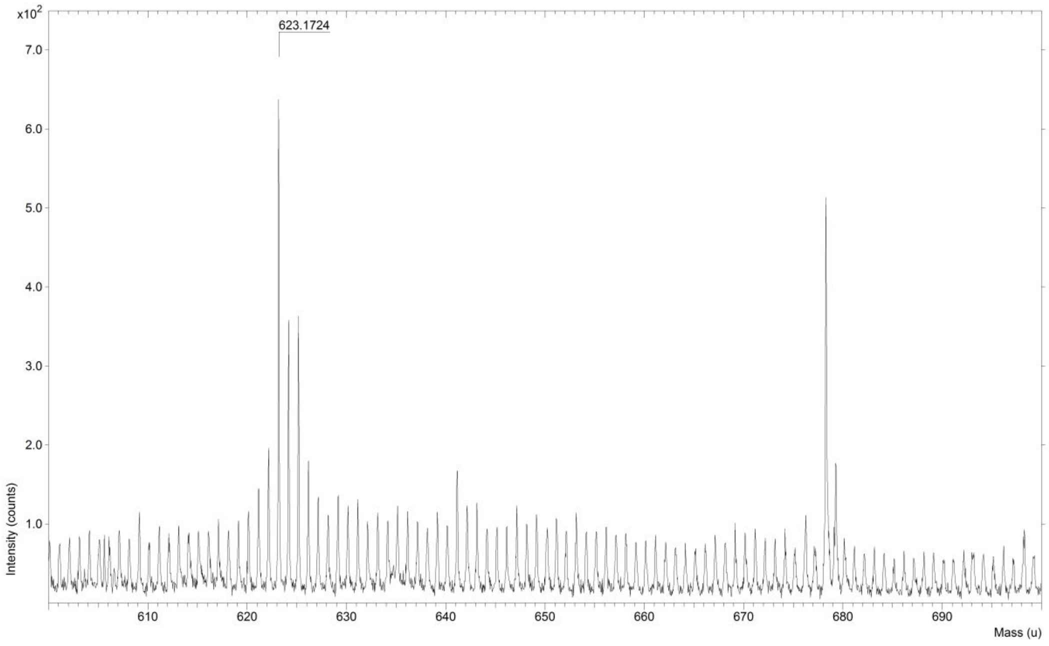

Figure S4. TOF-SIMS spectrum of **Ni(TTCorr)**.

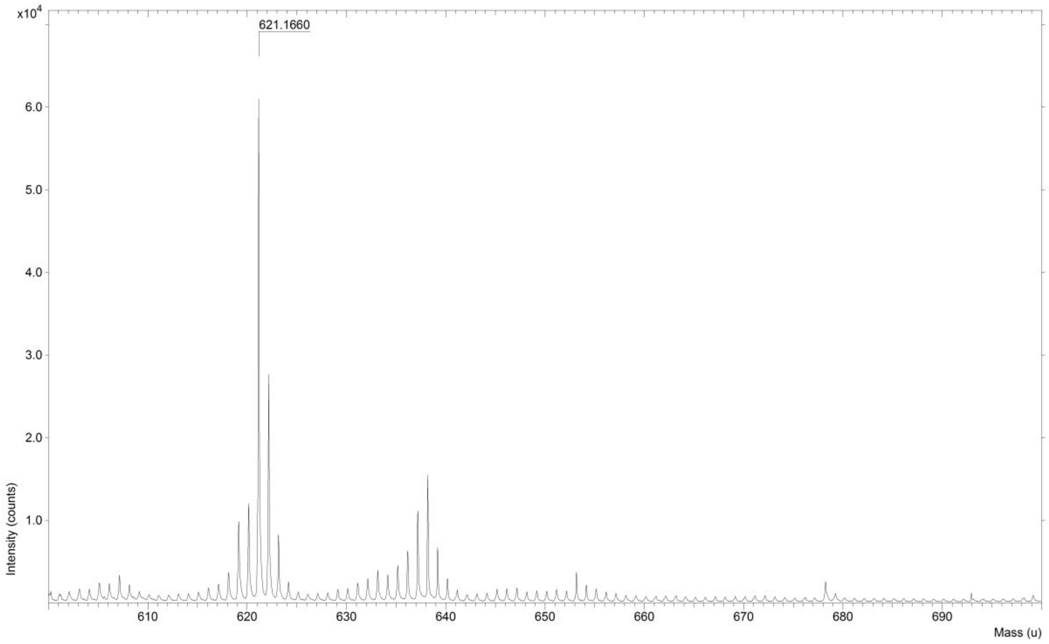

Figure S5. TOF-SIMS spectrum of **Fe(TTCorr)Cl**.

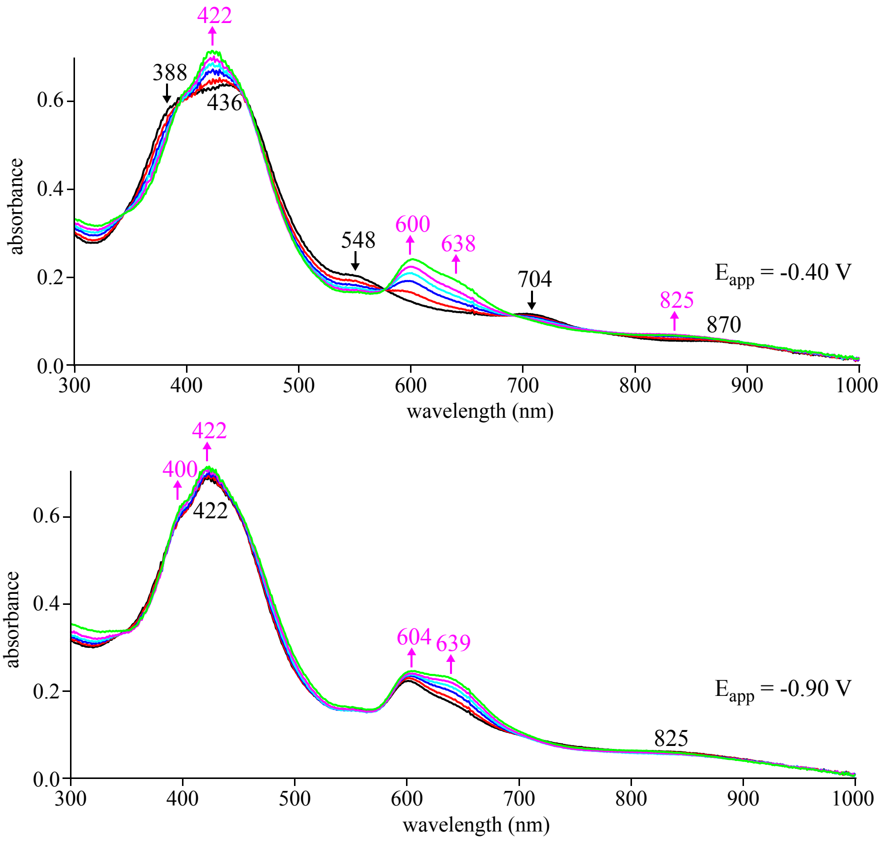

Figure S6. UV–vis spectral changes of Ni(TTCorr) during controlled potential reduction in CH_2Cl_2, 0.1 M TBAP at two different reducing potentials.

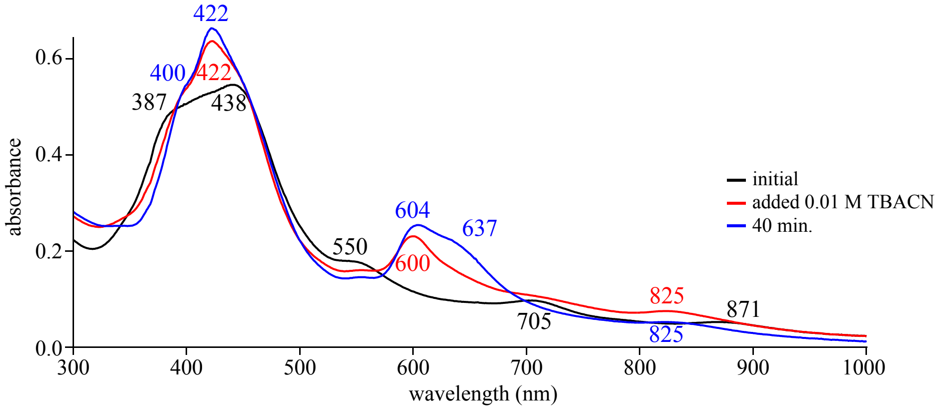

Figure S7. Ni(TTCorr) at ~10^{-5} M in CH_2Cl_2 before and after addition of cyanide ions in the form of TBACN.
